# Local floods induce large-scale abrupt failures of road networks

**DOI:** 10.1038/s41467-019-10063-w

**Published:** 2019-05-15

**Authors:** Weiping Wang, Saini Yang, H. Eugene Stanley, Jianxi Gao

**Affiliations:** 1 State Key Laboratory of Earth Surface Processes and Resource Ecology, Beijing Normal University, Beijing, 100875 PR China; 2 Key Laboratory of Environmental Change and Natural Disaster, Ministry of Education, Beijing, 100875 PR China; 3 Faculty of Geographical Science, Academy of Disaster Reduction and Emergency Management, Beijing Normal University, Beijing, 100875 PR China; 4 Center for Polymer Studies and Department of Physics, Boston University, Boston, MA 02215 USA; 5 Department of Computer Science, Rensselaer Polytechnic Institute, Troy, NY 12180 USA; 6 Network Science and Technology Center, Rensselaer Polytechnic Institute, Troy, NY 12180 USA

**Keywords:** Hydrology, Natural hazards, Complex networks, Statistical physics

## Abstract

The adverse effect of climate change continues to expand, and the risks of flooding are increasing. Despite advances in network science and risk analysis, we lack a systematic mathematical framework for road network percolation under the disturbance of flooding. The difficulty is rooted in the unique three-dimensional nature of a flood, where altitude plays a critical role as the third dimension, and the current network-based framework is unsuitable for it. Here we develop a failure model to study the effect of floods on road networks; the result covers 90.6% of road closures and 94.1% of flooded streets resulting from Hurricane Harvey. We study the effects of floods on road networks in China and the United States, showing a discontinuous phase transition, indicating that a small local disturbance may lead to a large-scale systematic malfunction of the entire road network at a critical point. Our integrated approach opens avenues for understanding the resilience of critical infrastructure networks against floods.

## Abstract (web summary)

The spread of flood-induced failures in critical infrastructure systems is understudied. Here the authors apply the CaMa-Flood global river flood simulation model to estimate the flood-induced failures and their spread in China and the US and find that the number of flood-induced total failures is in-between that of random and localized damage given the same intensity.

## Introduction

Increased greenhouse gas emissions due to human activity have resulted in an irreversible warming trend^1^. The additional moisture generated by global warming has increased precipitation worldwide, and floods have become more frequent^2–5^. Expanding populations and the exposure of assets in river basins and deltas have also increased the risk of future catastrophic floods^6,7^.

Recently, the spatiotemporal properties of floods have attracted researchers’ attention^8^ and encouraged the study of the impact of floods on critical infrastructure systems^9–18^. On the one hand, risk analyses have focused on methodologies of the road network absorption capacity^9^, hazard impact framework^19^, level of service remaining, and cascading effects within critical infrastructure systems^10,11^. These analyses help clarify the propagation of flood risk among infrastructure systems at different spatial scales^12^. On the other hand, since 2010, cascading failures on a network of networks^20,21^, sometimes called multilayer networks^22,23^ or multiplex networks^24^, have attracted more attention in network science. Relevant research has been motivated by the fact that diverse critical infrastructure systems, including transportation, power, energy, water, and communication systems, are coupled and depend on each other^21,25–29^. In interdependent networks, the failure of nodes in one network leads to the failure of dependent nodes in other networks, which in turn may cause further damage to the first network, leading to cascading failures and possibly catastrophic consequences^25^.

Percolation theory, based on statistical physics, allows analysis of the robustness of a network or a network of networks. In the percolation approach, network robustness is measured by the topological connectivity, the largest connected component, under the losses of links and nodes. In the case of transportation, an individual car can only travel along connected components; likewise, in the case of communication, a user can only send messages or emails along connected components. The largest connected component, also called the giant connected component, is thus assumed to be functional^30–32^. This measure is especially relevant to the robustness of a system, representing the function of mutual navigability under shocks^33^. The percolation approach was also shown to be extremely useful in addressing other scenarios, such as efficient damage and immunization^34–37^, in obtaining optimal paths^38^, and in designing robust networks^39^. Most studies, however, have focused on the network robustness against random^40^, localized^41^, and target damage^35,42^ rather than the network robustness against a realistic disturbance, such as a flood or earthquake.

**Fig. 1:**
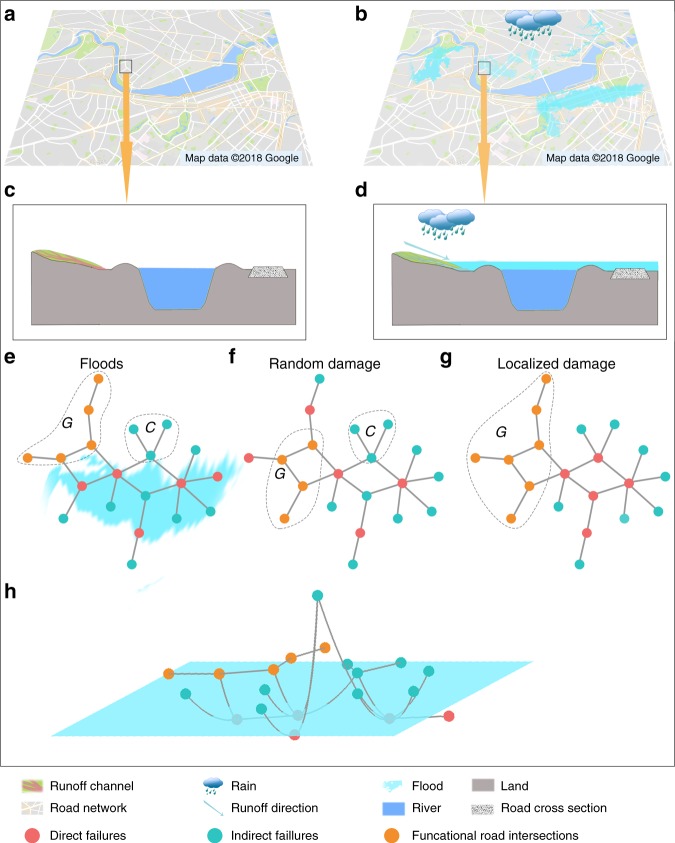
Schematic diagram of floods and demonstration of the effects of floods, random damage, and localized damage on a road system. **a** River and road network without flooding. **b** River and road network when rainfall-induced flooding inundates some road segments. **c** Sectional view of the boxed region in **a**. **d** Sectional view of the boxed region in **b**. The rainfall forms runoff on the ground. A large amount of runoff converges into a flood and inundates some road segments. The flood follows the river channel and damages infrastructure (e.g., roads) in a river basin. The flood-induced failures have a distinct trajectory. Schematic demonstrations of (**e**) random damage and (**g**) localized damage in a sketched network respectively. **f** 2D and **h** 3D views of flood disturbance in the sketched network

A flood, as a special and realistic type of disturbance, threatens the robustness of infrastructure systems^12,43,44^. As shown in Fig. 1, floods are locally destructive and the situation is similar to localized damage from this perspective. However, a flood can also affect the entire network owing to its wide spatial dispersion through rivers and the altitude of roads with a three-dimensional (3D) network structure, which is similar to the case of random damage. For example, risk analysis shows that local disruptions to infrastructure networks may have far-ranging effects in areas of indirect flooding^45^. Currently, limited research has focused on the structure and dynamics of 3D networks^46^. The currently used network-based framework, however, is unsuitable for 3D disturbances and 3D network topologies, where the altitude of a node or link, as the third dimension, crucially affects functionality. We thus still lack a systematic mathematical framework, such as percolation theory, with which to address the functionality of road networks under the disturbance of floods.

In this work, we develop a failure model to study the effect of floods on road networks and validate this failure model using historical data for Hurricane Harvey of 2017. We find that 90.6% of reported road closures and 94.1% of reported flooded streets are covered by the total failures obtained using the failure model and that the reported failures of each county in Houston strongly correlate with the coverage of total failures obtained using the failure model. We use the failure model to examine the effects of floods on the road networks of China and the United States (US) with millions of intersections and roads at varying altitudes. We simulate the flood using the CaMa-Flood global river flood model^8^ for given runoffs with three scenarios of flood distribution and obtain the direct failures of a road network measured by the inundated intersections. By applying the flood disturbance and percolation theory to road networks, we numerically study the properties of direct, indirect, and total failures due to flooding and compare them with those of random and localized damage. A flood is more locally destructive and has a stronger effect on the neighborhood or community than random damage and is different from localized damage because rivers may spread damage from one location to other locations. Surprisingly, direct failures are the major damage to road networks (as for localized damage) in the event of a small flood whereas indirect failures are the major damage (as for random damage) in the event of extreme floods. There is thus a discontinuous phase transition that significantly differs from the continuous phase transition observed in random and localized damage. Our findings reveal that at a critical point, a small local disturbance may lead to a large-scale systematic malfunction of the entire road network, allowing us to design effective strategies for preventing network-wide damage by identifying critical components that are the last line of defense against catastrophic abrupt collapses. These critical components have relatively high altitude and are closer to a river, in contrast to the measurements of degree, betweenness, and coreness centrality in classic two-dimensional (2D) networks. We finally use a message passing approach^47–50^ to develop an analytical solution for the effect of floods on road networks. These findings show that our integrated approach (of a failure model and analytical solution) opens avenues for understanding the robustness of 3D infrastructure networks against floods, with direct implications for risk reduction and management.

## Results

### Hurricane Harvey of 2017

We propose a failure model (see Methods for details) to study the effects of floods on road networks. Before we use this model, we take the catastrophic flooding in Houston and South East Texas due to Hurricane Harvey^51^ as a case study to validate the results of the proposed failure model. We collected the maximum observed flooding from Dartmouth Flood Observatory data sets^52^. After inputting the flood information to the actual US road network, we use our failure model to identify road intersections that have directly and indirectly failed (as shown in Supplementary Fig. 1). We collected information of road closures reported by TranStar and flooded streets (road segments) reported by public media^53^ in Houston and refer to these road closures and flooded streets as reported failures.

**Fig. 2:**
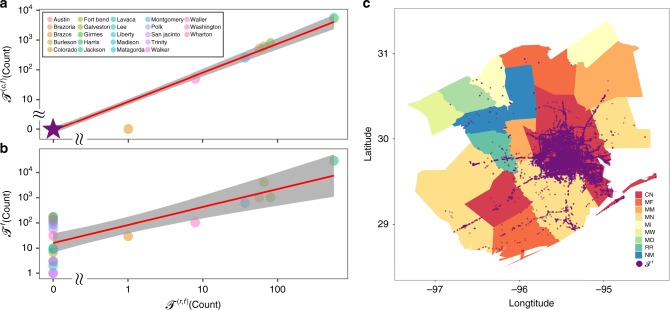
Comparison of realistic data and model results. **a** Size of ${\mathscr{T}}^{(c,f)}$ (the reported failures covered by total failures obtained using our failure model) as a function of the size of ${\mathscr{T}}^{(r,f)}$ (the reported failures) in different counties. **b** Size of ${\mathscr{T}}^{(f)}$ (total failures obtained using our failure model) as a function of the size of ${\mathscr{T}}^{(r,f)}$ in different counties. The purple star in **a** represents the sizes of ${\mathscr{T}}^{(r,f)}$ and ${\mathscr{T}}^{(c,f)}$ in 16 counties: Austin, Burleson, Colorado, Grimes, Jackson, Lavaca, Lee, Liberty, Madison, Matagorda, Polk, San Jacinto, Trinity, Walker, Washington, and Wharton. **c** Mapping of the description of flooding damage with total failures. Purple points in **c** represent ${\mathscr{T}}^{(f)}$

Comparing the total failures (road segments) identified by the proposed failure model with reported failures, we find that 90.625% of reported road closures and 94.10995% of reported flooded streets are covered by the total failures. ${\mathscr{T}}^{(r,f)}$ (the reported failures) strongly correlates with ${\mathscr{T}}^{(c,f)}$ (the reported failures covered by total failures) (correlation coefficient *r* = 1, as shown in Fig. 2a) and ${\mathscr{T}}^{(f)}$ (the total failures) (correlation coefficient *r* = 0.99, as shown in Fig. 2b).

We then use key words to categorize the damage descriptions in the region (see Methods for details) from the post tropical cyclone report of Hurricane Harvey issued by the National Oceanic and Atmospheric Administration (NOAA)^54^. Supplementary Table 1 lists the details of all nine categories, the number of totally failed nodes obtained using our failure model $|{\cal{N}}_i|$, and the ratio of failures in each category *i*, $F_i^{(t,f)}$. We find that the failure ratio is highly consistent with the category descriptions. For categories of catastrophic or major flooding with numerous roads inundated, the failure ratios are high, which shows the simulation results effectively reflect the actual pattern of flooding damage. The spatial distributions of total failures in counties are labeled using the nine categories, as shown in Fig. 2c. The spatial distribution of total failures obtained using our failure model is basically consistent with that of descriptions of flooding damage in the post tropical cyclone report.

With these comparisons, we can claim that the proposed model allows us to reasonably estimate the flood effect on a road system.

### Features of flood-induced failures

**Fig. 3:**
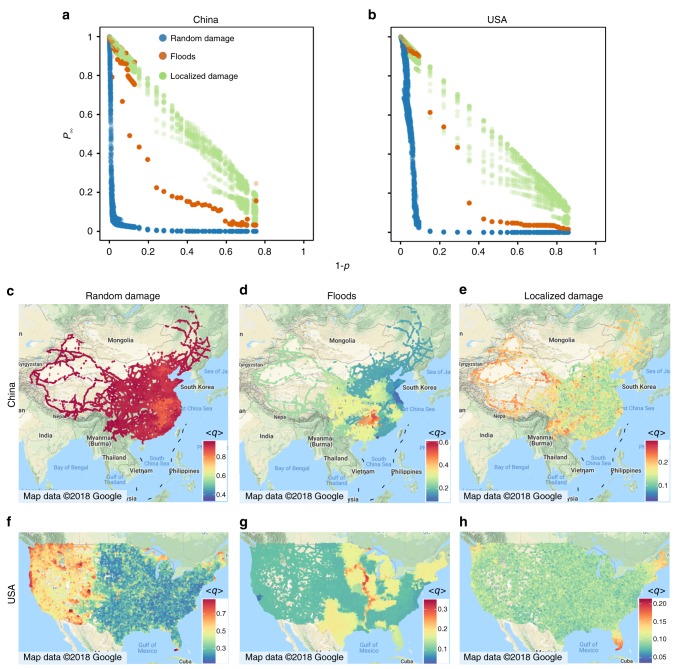
Features of flood-induced failures. Fraction of nodes in giant connected component (*P*_∞_) in China **a** and the US **b** as a function of the fraction of nodes that are direct failures (1 − *p*). Each road intersection is coded by color according to the vulnerability to total failure due to random damage (**c** ($\langle q_i^{(t,r)}\rangle$)), floods (**d** ($\langle q_i^{(t,f)}\rangle$)) and localized damage (**e** ($\langle q_i^{(t,l)}\rangle$)) in China. **f**–**h** Results for the US. The notations of aggregated vulnerability are given in Supplementary Table 2. We only consider road intersections in the giant connected component of the original road network. Simulation results are the outcome of 20 independent global simulation runs and 20 regional simulation runs

Floods are locally destructive and can affect an entire network, which is similar to the case for localized damage and random damage but there are unique features. To identify the features of flood-induced failures, we compare the effects of floods on a road network with those of random and localized damage (see Methods and Supplementary Note 1 for details) using the verified failure model. We apply three flood scenarios (see Methods for details) to produce various flood events and select the road networks of mainland China and the US for our national case study. Supplementary Fig. 2 shows the sizes of the giant connected components of Chinese and US road networks when disturbed by three different types of disturbance (i.e., flood events, random damage, and localized damage) with the same fraction of direct failures. We also select five flood-prone provinces in China and nine flood-prone states in the US as regional case study areas (see Supplementary Figs. 3 and 4). Figure 3 shows the national and regional simulation results for China and the US, revealing various characteristics of flood-induced failures. Figure 3a, b shows that the fraction of the giant connected component (*P*_∞_) in a road network after a flood (blue dotted line) lies between that of random damage (red line) and that of localized damage (green line) in all scenarios for both direct and indirect failures. This indicates that the effect of floods is between that of random damage and that of localized damage. This result suggests that floods are a new type of disturbance that has a unique damage pattern, which has rarely been studied in the network science literature.

We further explore the spatial distribution of flood-induced failure. Figure 3c–h and Supplementary Fig. 5 show an aggregated vulnerability map of road intersections under random damage, floods, and localized damage (see Methods for aggregated vulnerability and the notations of aggregated vulnerability are given in Supplementary Table 2). We obtain the following results for China.

The aggregated vulnerability of totally failed road intersections due to random damage ($\langle q_i^{(t,r)}\rangle$) is largest but is not spatially aggregated. The aggregate vulnerabilities of totally failed road intersections due to floods ($\langle q_i^{(t,f)}\rangle$) and localized damage ($\langle q_i^{(t,l)}\rangle$) are spatially aggregated but lower in value (see Fig. 3c–e).

The aggregated vulnerability of the direct failed road intersection due to flooding ($\langle q_i^{(d,f)}\rangle$) is largest and spatially aggregated [see Supplementary Fig. 5a–c].

The geographical distributions of the aggregated vulnerability of indirectly failed road intersections in China due to random damage ($\langle q_i^{(i,r)}\rangle$) and floods ($\langle q_i^{(i,f)}\rangle$) are similar to the distribution for direct failures, and the aggregated vulnerability of indirectly failed road intersections under random damage is larger than that of directly failed road intersections under random damage (see Supplementary Fig. 5a, b, d, e). The aggregated vulnerability of indirectly failed road intersections due to localized damage ($\langle q_i^{(i,l)}\rangle$) in China spatially aggregates but is smaller than that of directly failed road intersections ($\langle q_i^{(d,l)}\rangle$)(see Supplementary Fig. 5c, f).

Figure 3f–h and Supplementary Fig. 5g–l show similar results for the US but the geographical distributions of the aggregated vulnerability of indirect failures and total failures due to random damage in the US have two clearly separated areas, not one area as for China (see Fig. 3c, f and Supplementary Fig. 5d, j). This difference may be a result of regional simulations owing to random damage in the US (see Supplementary Fig. 4a) not totally destroying the network as they did in China (see Supplementary Fig. 3a).

We also examine the relationship between vulnerability and population density and find similar results (see Supplementary Fig. 6). The aggregated vulnerability of each population density to random-damage-induced total failures is the largest among the three types of disturbance, and the aggregated vulnerability is highest for flood-induced direct failures among the three types of disturbance.

Random damage thus causes the most destructive total failures, where the largest contribution is from indirect failures. All failures due to random damage are spatially randomly distributed, while the total failures due to floods and localized damage have geographical agglomerations. Direct failures make the largest contribution to flood-induced total failure, and indirect failures make the largest contribution to local damage-induced total failures.

### Percolation analysis

**Fig. 4:**
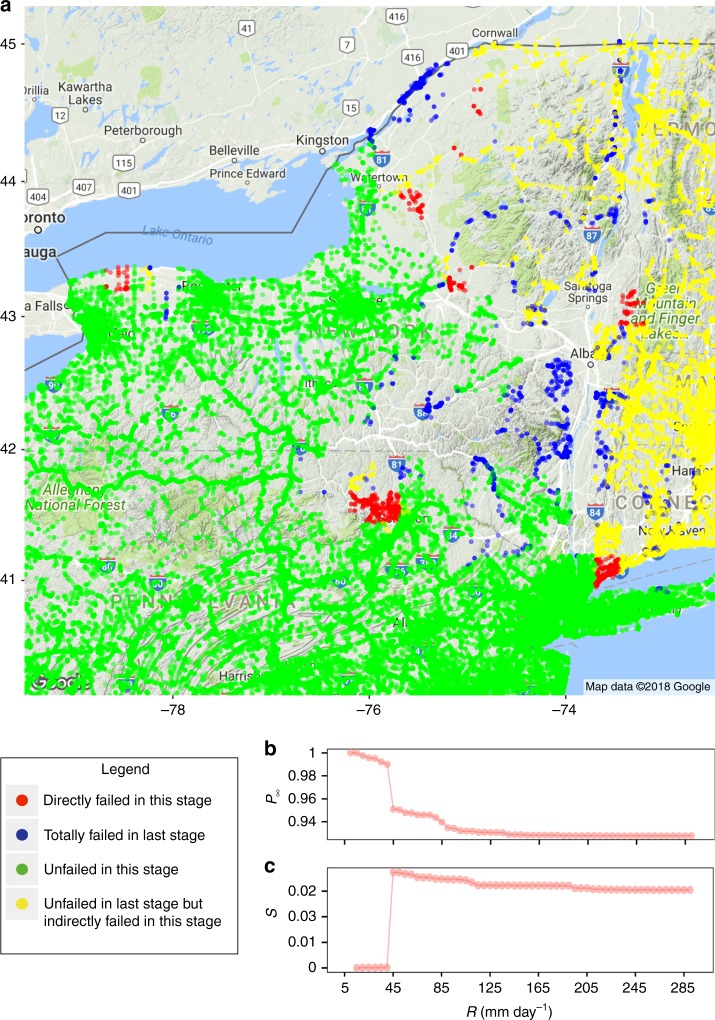
Percolation in New York. **a** Geographical layout of road intersections disturbed by flooding in New York under normal flooding scenarios when the input surface runoff (*R*) increases 40 mm (last stage) to 45 mm (this stage) per day. **b** Fraction of nodes in the giant connected component (*P*_∞_) of a road network in the US as a function of the runoff only in New York. **c** Fraction of nodes in the second largest connected component (*S*) on a road network of China as a function of the runoff only in New York

When comparing the effects of floods on a road system with the effects of random and localized damage, we discover that both China and US road networks show discontinuous phase transitions, indicating that a tiny increase in runoff may cause a large-scale systematic malfunction of the whole road network when the system is close to a critical point. Percolation phase transition occurs when the giant connected component disintegrates as the second largest connected component reaches its maximum^55^. The network of New York (Fig. 4b), for example, shows the phase transition for disturbance by floods. The simulation results indicate that the fraction of nodes in the second largest connected component (*S*) in the road network of the USA reaches a maximum value when the flood input runoff in New York (Fig. 4b) under a normal flood scenario increases from 40 to 45 mm per day. To clarify these potential critical points, we plot the geographical layout of road intersections disturbed by floods in New York (Fig. 4a) under a normal flood scenario. This set of newly failed (inundated) road intersections (red in Fig. 4a) is less destructive but is catastrophically destructive when combined with previously failed road intersections (blue in Fig. 4a). We observe similar percolation phenomena in other provincial case study areas; e.g., Sichuan (Supplementary Fig. 7). We compare four important measures (i.e., the degree, betweenness, altitude (DEM), and distance to a river) for the set of newly added inundated intersections (Vary) and that of all road intersections (Normal) in Sichuan, China when a phase transition emerges. Supplementary Fig. 8 shows that the newly inundated intersections at the critical point have higher altitude and are closer to a river. It is interesting to note that these intersections are not the traditionally recognized important components or critical components in a network, because their degree and betweenness values are small. However, they are the last line in a networks defense against being broken into disconnected components of subextensive size. These intersections should therefore be prioritized when developing strategies of disaster prevention; i.e., the road intersections that have higher altitude and are closer to a river are more crucial in flood mitigation.

### Comparison of numerical and analytical solutions

The present study uses failures obtained using the failure model to measure the effect of floods on a road network. To identify failures, we need to find the giant connected component of road networks under floods. Straightforwardly, using a numerical solution (see Methods for details), we can identify all nodes in the giant connected component of a network under flood but this may require much computer power and time. To alleviate this problem, we adopt a message passing approach^47–50^ and develop an analytical solution with which to compute the fraction of nodes in giant connected components *P*_∞_ under floods with a low computational cost. It is feasibly effective and helpful to derive the analytical solution for the effect of floods on a road network.

**Fig. 5:**
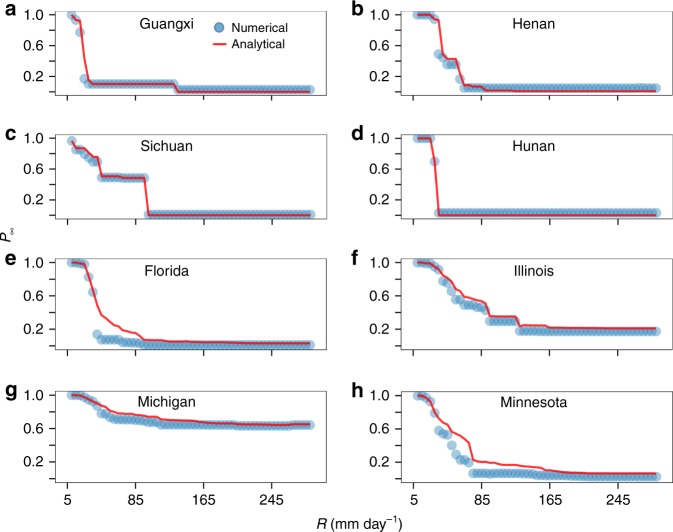
Comparison of numerical and analytical solutions. **a** The fraction of nodes in the giant connected component (*P*_∞_) of a road subnetwork in Guangxi as a function of surface runoff (*R*). Blue points represent the numerical results while the red line represents the analytical results. **b**–**h** Giant connected component for road subnetworks as a function of surface runoff

To test our analytical solution, we quantitatively compare simulation and analytical results. We extract the road intersections in a province from the national road network to form the initial subnetwork. We then obtain the final subnetwork by excluding the road intersections that are not in the giant connected component of the initial subnetwork. We apply this process to obtain the subnetworks for the other provinces and states. Figure 5 and Supplementary Fig. 9 show that our analytical results agree well with the giant component obtained from simulations. The analytical and simulation results are not perfectly consistent for Zhejiang, Florida, and New York because the networks in these regions are large (see Supplementary Table 3), have dense connections, and are no longer tree-like random systems.

## Discussion

The present paper introduces floods as a new type of disturbance in network science and explores the robustness of a road network against extreme floods. The aim of a road network, as part of critical infrastructure, is to facilitate the movement of people and goods and ensure economic activity. We develop a failure model with which to study the effects of floods on road networks. Coverage of 90.6% of reported road closures and 94.1% of reported flooded streets during Hurricane Harvey of 2017 validates the failure model. To quantify the relationship between the characteristics of floods and the different groups of induced failure (i.e., direct, indirect, and total), we use the failure model to study the characteristics of flood-induced failures and compare them with those of random and localized damage. We finally develop an analytical solution with which to calculate the effects of floods on road networks. Scientific findings of this work, such as road network percolation under a flood disturbance and a new type of important node in a road network, and their implications for reducing disaster risk are explored.

There are three unique aspects of flood-induced effects. First, the number of total failures due to floods lies between that due to random damage and that due to localized damage (as shown in Fig. 3a, b). Floods are more locally destructive and more strongly affect a neighborhood or community than random damage and are more globally destructive than localized damage because they can affect entire river basins. The destructive effect of floods is therefore between that of random damage and that of localized damage. This suggests that we may want to prioritize floods above most other natural hazards, e.g., earthquakes, which mainly conform to the features of localized damage. Second, the main contributor to total failures due to floods is direct failures while that to random damage and that to localized damage are indirect failures (as shown in Fig. 3c–h and Supplementary Fig. 5). This indicates that engineering and non-engineering measures of preventing direct failures are the best approaches of reducing flood damage. Third, a direct failure due to floods can destroy components that are considered more important (e.g., more populated nodes) and may affect more people than a direct failure due to other types of damage (Supplementary Fig. 6). Throughout human history, most major cities have been settled along rivers because rivers are vital to transportation and trade. Road systems, as the backbone of human activity and social development, are naturally concentrated in major river basins. It is thus expected that more-populated areas will be more affected by floods than by other hazards, which calls for more attention to be paid to the reduction of the disaster risk posed by floods.

More importantly, road networks in both China and the US show discontinuous phase transitions under a flood disturbance (Fig. 4 and Supplementary Fig. 7), which are different from continuous phase transitions that we observe under random damage and localized damage (Supplementary Figs. 3 and 4). This finding reveals that the road network is more vulnerable to a flood disturbance than to random or localized damage. The reason is that for a continuous phase transition, a small perturbation can only result in a small damage; i.e., $\frac{{dP_\infty }}{{dp}}$ is finite. However, for discontinuous phase transition, a small perturbation can cause much damage; i.e., $\frac{{dP_\infty }}{{dp}}$ is infinite at a critical point. It is essential to predict the threshold of the discontinuous phase transition, because even when the giant connected component is large, a small fraction of direct failures may cause the large-scale collapse of the entire system. For the same fraction of node removal, random damage may cause more damage to a road network than a flood, but their damage is predictable because of the continuous phase transition. It is thus important to analyze the percolation function and predict the critical threshold. We analyze the percolation phenomena adopting the message passing approach by solving a set of equations. The analytical results are highly consistent with the simulation results. While the computation time of searching for the giant component from the large-scale road network with multiple types and numerous possibilities of disturbances may be hopelessly long, the time required to compute the analytical solution of the threshold is much shorter. We can therefore analyze the robustness of the road network for all possible scenarios of floods and identify the critical intersections/roads, allowing us to better reduce risks to infrastructure by protecting the critical intersections/roads and designing quantitative analysis tools for an early warning system, and providing decision-making support in infrastructure planning and design.

We also note that some nodes that have low centrality (i.e., low degree and betweenness values) but higher altitude and are closer to a river, seemingly unimportant in a 2D network, are the last line of defense against a collapse of the whole network into several smaller connected components (see Fig. 4, Supplementary Figs. 7 and 8). This finding contrasts with the common understanding and existing disaster risk reduction practices, which tend to focus and invest much more on nodes considered important with higher centrality. Why the unimportant nodes become the last straw that breaks the camels back and how these nodes combine with damage patterns to trigger abrupt systematic malfunctions call for more research in the broad field of disaster risk science. Comparing the affected population between China and the US (see Supplementary Fig. 10 and Supplementary Note 2 for details), we find that more highly populated counties are affected by floods more in China than in the US; counties with an extremely high population density ([95, 100] percentile) are more likely to be directly affected by floods in China; and more people are indirectly affected by floods in China. All these findings indicate that flood mitigation will be more challenging in China than in the US.

Floods are typical 3D disturbances. The coupling of floods with infrastructure systems, which are usually 3D, will create unique damage patterns. For example, road segments in a low-lying area will be submerged by water while those at high altitude may remain intact in the event of flooding. The coupling may also contribute to waterlogging after storms, a problem with which many modern cities are struggling. The mechanism of this coupling of two 3D systems should be explored in future research.

When networked critical infrastructure systems, such as transportation systems and power grids, are disturbed by floods, knowing the temporal-spatial properties of direct, indirect, and total failures is essential for risk assessment and reduction. If we find universal features of failures due to floods, we can use them to optimize the planning and design of networked critical infrastructure systems. The proposed integrated approach that we have applied to floods can also be applied to other realistic spatial disturbances, including tropical cyclones, tornadoes, and debris flows.

## Methods

### Data and pre-processing

All data sets used in this analysis are publicly available. The boundary of study areas in China and the US are respectively collected from the National Geomatics Center of China (http://www.ngcc.cn/) and United States Census Bureau (https://www.census.gov/geo/maps-data/data/cbf/cbf_counties.html). The digital elevation model (DEM) and the river network of the study areas are extracted from HydroSHEDS (https://hydrosheds.cr.usgs.gov/). The population density is taken from the Gridded Population of the World, Version 4 (GPWv4) (http://sedac.ciesin.columbia.edu/data/collection/gpw-v4). The maximum observed flooding caused by Hurricane Harvey is collected from the Dartmouth Flood Observatory data sets (http://floodobservatory.colorado.edu/Events/2017USA4510/2017USA4510.html). We collect information of road closures reported by TranStar and flooded streets (road segments) via public media^53^ in Houston from https://www.chron.com/news/houston-weather/hurricaneharvey/article/These-are-the-roads-that-are-closed-in-Houston-12003482.php.

We select five types of major road and four types of highway link from OpenStreet data sets (https://www.openstreetmap.org) as our road data sets (as shown in Supplementary Table 4). After importing these road data sets (in shapefile format), we use geographical information system software including ArcGIS to obtain the starting point and ending point of each road segment. After removing duplicates, these starting and ending points can be seen as road intersections and are abstracted as nodes. We build an edge between two nodes if and only if one of the nodes is the starting point of a road segment and the other node is the ending point of the same road segment. We finally obtain the road network to represent the road system. We thus use networks to represent road systems in China and the US, after representing road intersections as nodes and road segments as edges. The road network in China contains 1,143,837 nodes and 944,600 major links, and its giant component has 329,802 nodes. The US road network contains 2,231,327 nodes and 2,191,289 links, and its giant component has 1,590,077 nodes. To ensure the connectivity of the entire road system, we use the giant connected component of a road network to represent the road system.

We use the CaMa-flood model to simulate flood events. The source codes, executable scripts, global river map, and regionalization tools for the CaMa-flood model are available at http://hydro.iis.u-tokyo.ac.jp/yamadai/cama-flood/index.html. The methods of producing input runoffs for the CaMa-flood model are described in Methods and MATLAB code for producing these input runoffs is available at a GitHub repository (https://github.com/wpwang90/IAERCURNetworks). We obtain a flood inundation depth map from the CaMa-flood outputs and use the ArcGIS extract tool to extract the inundation depth of road nodes from this inundation depth map. The MATLAB code for format conversion from the binary file resulting from the CaMa-flood model to GeoTIFF is available at a GitHub repository (https://github.com/wpwang90/IAERCURNetworks).

We use geodata from Google Maps to map the road nodes in our study areas after calling functions from ggmap (an R package)^56^.

### Flood simulation

Runoff is the rainfall that falls to the surface and flows on the surface or underground under the action of gravity. The ratio of surface runoff to rainfall has a different empirical value for different land cover types. We thus use surface runoff to represent the rainfall. Inputting different surface runoffs representing different degrees of rainfall, we use the CaMa-Flood^57^ global river model (see Supplementary Note 3 for details) to simulate floods at different intensities. In this paper, we propose three types of flood scenario to simulate floods as follows.

### Normal flood scenarios

Surface runoff is the same everywhere within the study area. To produce floods at different intensities, we set the surface runoff from 1 to 9 mm per day with a step size of 1 and from 10 to 300 mm per day with a step size of 5 mm. We finally have 68 unique runoff scenarios that induce 68 flood events in the study area.

### Random flood scenarios

To produce floods at different intensities, we set the reference surface runoff from 20 to 300 mm per day with a step size of 5 mm. For each position in the study area, we choose a reference surface runoff minus a uniform random value, which ranges from 0 to 20 mm, as the actual surface runoff. We thus have 57 unique runoff scenarios. To reduce the effect of random numbers, we independently repeat this process 20 times. We finally have 1140 unique runoff scenarios that induce 1140 flood events in the study area.

### Pearson flood scenarios

We set the reference surface runoff from 20 to 300 mm per day with a step size of 5 mm. For each position in the study area, we choose a reference surface runoff minus a Pearson-III random number, which ranges from 0 to 20 mm, as the actual surface runoff. We thus have 57 unique runoff scenarios. To reduce the effect of random numbers, we independently repeat this process 20 times. We finally have 1140 unique runoff scenarios that induce 1140 flood events in the study area.

We select the road networks of mainland China and the US to be our national simulation cases and five flood-prone provinces in China and nine flood-prone states in the US as regional simulation cases. Applying the three types of flood scenario in these study areas, we have a total of 37,568 unique flood events.

### Failure model

A road system consists of many road segments and intersections. We can use a network to represent the road system, after representing intersections as nodes and road segments as edges. In this study, we use the size of failures (i.e., the number of failed nodes) to measure the effect of floods on road systems. We categorize road intersection failures due to disturbances into three groups: a direct failure, where the road intersection is located in the flooded area and is lower than the water surface, an indirect failure, where the road intersection cannot maintain traffic flow because it is disconnected from the giant connected component of the road system after some road intersections undergo direct failure, and a total failure, which is a direct failure or indirect failure.

### Comparing floods with random damage and localized damage

Because a flood has three-dimensional features, it is crucial to explore the common and specific characteristics among a flood disturbance and well-studied random damage and localized damage. The direct failures resulting from these three types of disturbance are as follows.

For a flood disturbance, we use the CaMa-Flood global river model^8^ to simulate the inundation depth in an area after inputting a runoff scenario. In this study, three types of runoff scenario are used to represent a multitude of flood events (see Methods). Each flood event has a unique distribution of the inundation depth depending on the DEM of the area. The altitudes of the water surface and road intersections are compared, and those road intersections (nodes) lower than the water surface are considered to have directly failed.

For a random damage, we randomly remove road intersections from the entire network. To compare the damage effect between a pair of a flood disturbance and random damage on a road network, we count the number of direct failures due to flood disturbance and generate the same number of removed nodes in the paired random damage. These removed nodes are direct failures due to paired random damage.

For a localized damage, we remove road intersections by adopting a random walk starting from a random node (intersection) in the network. To compare the damage effect between a pair of a flood disturbance and localized damage on a road network, we count the number of removed nodes due to flood disturbance and generate the same number of removed nodes in the paired localized damage. These removed nodes are direct failures due to paired localized damage.

As shown in Fig. 1, we use this sketch network ($|{\cal{N}}| = 20$) to compare the failures resulting from floods with those resulting from random damage and localized damage on the network. The nodes are removed as a result of any type of disturbance; i.e., the red nodes in Fig. 1e–g. We refer to these removed nodes as direct failures ${\cal{D}}$. When one flood event occurs, we acquire the inundated nodes (direct failures) in the road network, remove them, and its size is $|{\cal{D}}| = 5$ (Fig. 1e). When comparing the effect of this flood event with the effects of random damage and localized damage, we set the fraction of direct failures in total nodes, $1 - p = \frac{1}{4}$, for all three types of disturbance in this example. After removing these nodes (direct failures), some nodes are disconnected from the giant connected component of the road system (i.e., the majority of the road system). This means the vehicles on these nodes cannot reach the majority of nodes in a network. These nodes are referred to as indirect failures and the set of these nodes is denoted ${\cal{I}}$. In the example of Fig. 1, we find the fractions of indirect failures among all nodes are $\frac{{11}}{{20}}$, $\frac{1}{2}$, and $\frac{2}{5}$, and the network breaks into three distinct connected components (${\cal{C}}$), two distinct connected components (${\cal{C}}$ & ${\cal{P}}$), and only one connected component (${\cal{P}}$) for random damage, floods, and localized damage respectively. The joint set of direct and indirect failures is referred to as the total failures ${\mathscr{T}}$, and our order parameter, $P_\infty = \frac{{|{\cal{P}}|}}{{|{\cal{N}}|}}$, is the fraction of nodes in the giant component. When comparing floods with random damage, we generate random damage according to the results of each flood event. When a flood event occurs, some nodes will be inundated and removed from the road network. For a corresponding random damage event, we randomly remove the same number of nodes from the road network as in a flood event. Owing to the randomness of simulation, we generate 20 random damage events for one flood event. We then calculate and compare the sizes of the giant connect component under this flood event and random damage. The comparison of floods and localized damage is performed in a similar manner.

### Failures resulting from Hurricane Harvey

To validate the failure model, we classify five groups of failure as follows.

Reported failures. We collect information of road closures reported by TranStar and flooded streets (road segments) via public media^53^ for the flood in Houston and refer to these as reported failures (${\mathscr{T}}^{(r,f)}$).

Direct failures. In this empirical study, we do not have the inundation depth and we therefore simply remove nodes in a flooded area from the network and refer to them as direct failures.

Indirect failures. By removing direct failures resulting from flooding, some nodes or connected components may become disconnected from the new giant connected component; these are referred to as indirect failures (${\cal{I}}^{(f)}$).

Total failures. The total failures (${\mathscr{T}}^{(f)}$) resulting from floods refer to the combination of direct and indirect failures; i.e., ${\mathscr{T}}^{(f)} = {\cal{D}}^{(f)}{\bigcup} {{\cal{I}}^{(f)}}$.

Reported failures covered by total failures. For a reported failure, if there exists a total failure within a geographical distance of 10 meters, we deem that this reported failure is covered by the total failures in the context of a flood. The set of these reported failures covered by total failures is denoted ${\mathscr{T}}^{(c,f)}$.

### Categorization of descriptions of damage

The post tropical cyclone report issued by NOAA (NOAA, 2018) provides descriptions of flooding damage for the majority of counties in Houston, Texas. We categorize these descriptions into nine groups by key words. For instance, the damage description for the county of Colorado is as follows.

*“Major flooding occurred along the Colorado River with widespread inundation of crop land near Eagle Lake, as well as numerous roads inundated included US HWY90, FM950 and lowest homes flooded in the vicinity of the gauge or in the floodplain. Colorado overtops its levees around 47 feet from Columbus down to Bay City, causing extensive flooding in low lying areas along the left bank.”*

We abstract the damage description for the county of Colorado using the key words major flooding occurred and numerous roads inundated and then categorize this county into group MN in Supplementary Table 1. We define the ratio of total failures resulting from floods ($F_i^{(t,f)}$) in a county *i* as

1 $$F_i^{(t,f)} = \frac{{|{\mathscr{T}}_i^{(f)}|}}{{|{\cal{N}}_i|}},$$

where ${\cal{N}}_i$ and ${\mathscr{T}}_i^{(f)}$ respectively denote the sets of nodes and totally failed nodes obtained using our failure model in county *i*.

### Percolation theory

A connected component ${\cal{C}}$ in a network is a subnetwork in which any nodes can be connected to the others by paths, which can be represented by a sequence of connected edges. The giant connected component is the connected component that contains the largest fraction of the whole network. Percolation theory has been introduced to study network stability and predicted the critical percolation threshold. The robustness of a network is usually either characterized by the value of the critical threshold analyzed using percolation theory or defined as the integrated size of the giant connected component during the entire damage process^21^. When the fraction of removed nodes (direct failures) reaches a certain value 1 − *p*_*c*_, there is a percolation phase transition where the whole system will completely fragment and lose its function. This critical (percolation) threshold *p*_*c*_ indirectly reflects the robustness of the network. Phase transition emerges when a tiny change in a state variable of a system causes an abrupt change in some properties of the system; e.g., the vaporization of water, superconductivity in metals, and the spreading of epidemic disease. The order of the phase transition can be specified from whether the macroscopic statistical property changes continuously (second order) or discontinuously (first order) at the transition. We also refer to this drastic change in some properties of the network system as percolation^58^. The system percolation in different disturbance scenarios has been widely studied^25,28,30–32,34,55,58–69^.

### Aggregated vulnerability

We quantify the vulnerability of a road intersection (node) *i* using probability *q*_*i*_, which is the probability of failure of a road intersection when disturbed. The aggregated vulnerability 〈*q*_*i*_〉 is the average of the failure probability of a simulated road intersection and is expressed as

2 $$\langle q_i\rangle = \frac{{\mathop {\sum}\limits_j {q_{i,j}} }}{{\mathop {\sum}\limits_j 1 }},$$

where *i* is the indicator of simulation runs while *q*_*i*,*j*_ is the probability that road intersection *i* fails during simulation *j*.

### Numerical solution

The giant connected component ${\cal{P}}$ is the largest connected component. This measure is especially relevant to the reliability of a road system in that it represents the ability of the system to remain navigable in the face of failures. We use the fraction of nodes in the giant connected component of a road network after disturbance to measure the effect of a disturbance on a road network. Straightforwardly, when a flood disturbance affects a road network, some nodes are removed from the road network and we can use either a breadth-first search or depth-first search to identify all nodes in the connected components of a network. In this study, we use the following depth-first algorithm^70^ to calculate the connected component for each node *v* (${\cal{C}}_v$).

Step 1. Choose a node *v* in a network, identify all its adjacent nodes (denoted *Q*), and set the connected component of ${\cal{C}}_v$ as empty (${\cal{C}}_v = \emptyset$).

Step 2. Pop a node from *Q*, add it to ${\cal{C}}_v$, and add all its adjacent nodes to *Q*.

Step 3. Repeat Step 2 until *Q* is empty.

### Analytical solution

We also use our theoretical model to compute giant connected components. In other words, the fraction of nodes in the giant connected component *P*_∞_ can be obtained by searching through simulation, which may require much computer power and time. The size of the giant connected component can also be obtained using equations.

A network is a set of nodes ${\cal{N}}$ connected by edges ${\cal{M}}$. The vector ${\mathbf{n}} = (n_1,...,n_{|{\cal{N}}|})^{T}$ represents which nodes have and have not failed. The vectors ${\mathbf{e}} = (e_1,...,e_{|{\cal{M}}|})^{T}$, ${\mathbf{o}} = (o_1,...,o_{|{\cal{M}}|})^{T}$, and ${\mathbf{d}} = (d_1,...,d_{|{\cal{M}}|})^{T}$ respectively represent the edge, starting node, and ending node of an edge. A given node *i* can be disconnected from the giant connected component ${\cal{P}}$, either because it has directly failed or because it has been indirectly detached via the failure of other nodes. The variable *v*_*i*_ is the probability of node *i* belonging to the giant connected component, *v*_*i*_ = 1 if $i \in {\cal{P}}$, *v*_*i*_ = 0. *v*_*ij*_ = 1, 0 represents the probability of *i* belonging to the giant connected component in the absence of *j*. The fraction of nodes in the giant component under direct failure condition *n* is then given by

3 $$P_\infty ({\mathbf{n}}) = \frac{{\mathop {\sum}\limits_{i = 1}^{|{\cal{N}}|} {v_i} }}{{|{\cal{N}}|}}.$$

For a real loopy network (strictly valid for the locally tree-like random network), we have the message passing formula^47–50,61^

4 $$v_{i,j} = n_i[1 - \mathop {\prod}\limits_{k \in \partial i \setminus j} {(1 - v_{k,i})} ],$$

where ∂*i*/*j* is a set of nearest neighbors of *i* minus *j*. We can finally put *j* back in the network and get the value of *v*_*i*_ as

5 $$v_i = n_i[1 - \mathop {\prod}\limits_{k \in \partial i} {(1 - v_{k,i})} ].$$

The Eq. (4) can be rewritten as

6 $$v_{e_l} = n_{o_l}[1 - \mathop {\prod}\limits_{k = 1}^{|{\cal{M}}|} {(1 - v_{e_k})^{B_{e_k,e_l}}} ],$$

where

7 $$B_{e_k,e_l} = \left\{ {\begin{array}{*{20}{c}} 1 & {if{\mkern 1mu} d_k = = o_l{\mkern 1mu} and{\mkern 1mu} o_k \ne d_l} \\ o & {else} \end{array}} \right.$$

The Eq. (6) can be rewritten as

8 $$v_{e_l} = n_{o_l}[\mathbf{1} - e^{\mathop {\sum}\limits_{k = 1}^{|{\cal{M}}|} {B_{e_k,e_l}ln(\mathbf{1} - v_{e_k})} }].$$

We write vector ${\mathbf{V}} = {(v_{e_1},v_{e_2},...,v_{e|{\cal{M}}|})}^{T}$, matrix ${\cal{B}} = |B_{e_i,e_j}|_{|{\cal{M}}| \times |{\cal{M}}|}$ (known as a non-backtracking matrix), **1**=(1, 1,..., 1)^*T*^_|*M*|_, and vector ${\mathbf{N}}_E = (n_{o_{e_1}},n_{o_{e_2}},...,n_{o_{e_{|{\cal{M}}|}}})^{T}$, and use circle to indicate Hadamard product, and Eq. (8) can then be rewritten as

9 $${\mathbf{V}} = {\mathbf{N}}_E\circ[\mathbf{1} - e^{{\cal{B}}ln(\mathbf{1} - {\mathbf{V}})}].$$

After solving Eq. (9) and putting the solution **V** back into Eq. (5), we use Eq. (3) to find the giant connected component under failure condition **n**.

We propose the following algorithm to solve Eq. (9).

Step 1. Initialize the vector **V** and direct failure condition **n**. A random number *v*_0,*i*_ ~ U(0, 1) is placed on each edge i of the spatial network (denoted ${\mathbf{V}}_0 = (v_{o,1},v_{o,2},...,v_{o,|{\cal{M}}|})^{T}$. We obtain the value of **N**_*E*_ according to direct failure condition *n*.

Step 2. Update the vector **V**. We calculate ${\mathbf{V}} = {\mathbf{N}}_E\circ[\mathbf{1} - e^{{\cal{B}}ln(\mathbf{1} - {\mathbf{V}}_0)}]$ using Eq. (9).

Step 3. Check for accuracy. If |**V** − **V**_0_| < *δ* (where we set *δ* = 0.0001 in this study), go to Step 4; else set **V**_0_ = **V** and go to Step 2.

Step 4. Calculate *v*_*i*_. Put the solution **V** back into Eq. (5) and obtain the value of *v*_*i*_.

Step 5. Calculate *P*_∞_(**n**). Using Eq. (3), we obtain the value of the fraction of nodes in the giant component under direct failure condition **n**.

## Supplementary information

Supplementary Information

## Data Availability

The data sets are publicly available and are open access online as stated in Data and preprocessing. All data sets are available from the authors upon reasonable request.

## Code availability

The source codes, executable scripts, regionalization tools for CaMa-flood model are available at http://hydro.iis.u-tokyo.ac.jp/yamadai/cama-flood/index.html. The data generation and/or analyses in this study are coded in R and/or MATLAB and are available at a GitHub repository (https://github.com/wpwang90/IAERCURNetworks).

## References

[1] IPCC. Summary for policymakers. In *Climate Change 2014: Impacts, Adaptation, and Vulnerability. Part A: Global and Sectoral Aspects. Contribution of Working Group II to the Fifth Assessment Report of the Intergovernmental Panel on Climate Change* (eds. Field, C. et al.) 33 (Cambridge University Press, Cambridge, 2013).

[2] Frei C, Schöll R, Fukutome S, Schmidli J, Vidale PL (2006). Future change of precipitation extremes in europe: Intercomparison of scenarios from regional climate models. J. Geophys. Res.: Atmosp..

[3] Christensen JH, Christensen OB (2007). A summary of the prudence model projections of changes in european climate by the end of this century. Clim. Change.

[4] Fowler H, Ekström M (2009). Multi-model ensemble estimates of climate change impacts on uk seasonal precipitation extremes. Int. J. Climatol..

[5] Nikulin G, Kjellström E, Hansson U, Strandberg G, Ullerstig A (2011). Evaluation and future projections of temperature, precipitation and wind extremes over europe in an ensemble of regional climate simulations. Tellus A.

[6] Dankers, R. & Feyen, L. Climate change impact on flood hazard in europe: an assessment based on high-resolution climate simulations. *J. Geophys. Res.: Atmosph.* **113**, D19105 (2008).

[7] Whitfield P (2012). Floods in future climates: a review. J. Flood Risk Manag..

[8] Hirabayashi Y (2013). Global flood risk under climate change. Nat. Clim. Change.

[9] Lhomme S, Serre D, Diab Y, Laganier R (2013). Analyzing resilience of urban networks: a preliminary step towards more flood resilient cities. Nat. Hazards Earth Syst. sciences.

[10] Pescaroli G (2018). Perceptions of cascading risk and interconnected failures in emergency planning: Implications for operational resilience and policy making. Int. J. Disaster Risk Red..

[11] Kelman I (2018). Connecting theories of cascading disasters and disaster diplomacy. Int. J. Disaster Risk Red..

[12] Serre D, Heinzlef C (2018). Assessing and mapping urban resilience to floods with respect to cascading effects through critical infrastructure networks. Int. J. Disaster Risk Red..

[13] Serre D (2018). DS3 Model Testing: Assessing Critical Infrastructure Network Flood Resilience at the Neighbourhood Scale.

[14] Serre D, Barroca B, Balsells M, Becue V (2018). Contributing to urban resilience to floods with neighbourhood design: the case of am sandtorkai/dalmannkai in hamburg. Journal of Flood Risk Management.

[15] Zevenbergen C (2018). Assessing quick wins to protect critical urban infrastructure from floods: a case study in bangkok, thailand. Journal of Flood Risk Management.

[16] Bambara G, Peyras L, Felix H, Serre D (2015). Developing a functional model for cities impacted by a natural hazard: application to a city affected by flooding. Natural hazards and earth system sciences.

[17] Bambara G, Peyras L, Felix H, Serre D (2014). Developing a performance evaluation functional model for cities impacted by a natural hazard: application to a city affected by flooding. Natural Hazards and Earth System Sciences Discussions.

[18] Serre, D. et al. Assessing vulnerability to floods of the built environment-integrating urban networks and buildings. In *Vulnerability, Uncertainty, and Risk: Analysis, Modeling, and Management*, 746–753 (2011).

[19] Hereld, M. & Kim, K. Disaster = infrastructure + hazard. In *2017 Resilience Week (RWS)*, 170–176 (2017).

[20] Gao J, Buldyrev SV, Havlin S, Stanley HE (2011). Robustness of a Network of Networks. Phys. Rev. Lett..

[21] Gao J, Buldyrev SV, Stanley HE, Havlin S (2012). Networks formed from interdependent networks. Nat. Phys..

[22] Danziger MM, Bonamassa I, Boccaletti S, Havlin S (2019). Dynamic interdependence and competition in multilayer networks. Nat. Phys..

[23] De Domenico M, Granell C, Porter MA, Arenas A (2016). The physics of spreading processes in multilayer networks. Nat. Physi..

[24] Osat S, Faqeeh A, Radicchi F (2017). Optimal percolation on multiplex networks. Nat. Commun..

[25] Buldyrev SV, Parshani R, Paul G, Stanley HE, Havlin S (2010). Catastrophic cascade of failures in interdependent networks. Nature.

[26] Helbing D (2013). Globally networked risks and how to respond. Nature.

[27] Committee on Climate Change. UK Climate Change Risk Assessment 2017–Synthesis report: priorities for the next five years. Tech. Rep., Committee on Climate Change (2010).

[28] Gao J, Li D, Havlin S (2014). From a single network to a network of networks. Natl. Sci. Rev..

[29] Wang W (2018). An approach for cascading effects within critical infrastructure systems. Phys. A: Stat. Mech. Appl..

[30] Yuan X, Hu Y, Stanley HE, Havlin S (2017). Eradicating catastrophic collapse in interdependent networks via reinforced nodes. Proc. Natl. Acade. Sci. USA.

[31] Parshani R, Buldyrev SV, Havlin S (2011). Critical effect of dependency groups on the function of networks. Proc. Natl. Acad. Sci. USA.

[32] Reis SD (2014). Avoiding catastrophic failure in correlated networks of networks. Nat. Phys..

[33] Kitsak M (2018). Stability of a giant connected component in a complex network. Phys. Rev. E.

[34] Callaway DS, Newman ME, Strogatz SH, Watts DJ (2000). Network robustness and fragility: percolation on random graphs. Phys. Rev. Lett..

[35] Cohen R, Erez K, Ben-Avraham D, Havlin S (2001). Breakdown of the internet under intentional attack. Phys. Rev. Lett..

[36] Cohen R, Havlin S, Ben-Avraham D (2003). Efficient immunization strategies for computer networks and populations. Phys. Rev. Lett..

[37] Chen Y, Paul G, Havlin S, Liljeros F, Stanley HE (2008). Finding a better immunization strategy. Phys. Rev. Lett..

[38] Braunstein LA, Buldyrev SV, Cohen R, Havlin S, Stanley HE (2003). Optimal paths in disordered complex networks. Phys. Rev. Lett..

[39] Schneider CM, Moreira AA, Andrade JS, Havlin S, Herrmann HJ (2011). Mitigation of malicious attacks on networks. Proc. Natl. Acad. Sci..

[40] Albert R, Jeong H, Barabási AL (2000). Error and attack tolerance of complex networks. Nature.

[41] Shao S, Huang X, Stanley HE, Havlin S (2015). Percolation of localized attack on complex networks. N. J. Phys..

[42] Huang X, Gao J, Buldyrev SV, Havlin S, Stanley HE (2011). Robustness of interdependent networks under targeted attack. Phys. Rev. E.

[43] Ganin AA (2017). Resilience and efficiency in transportation networks. Sci. Adv..

[44] Hong L, Ouyang M, Peeta S, He X, Yan Y (2015). Vulnerability assessment and mitigation for the chinese railway system under floods. Reliab. Eng. Syst. Saf..

[45] Hummel MA, Berry MS, Stacey MT (2018). Sea level rise impacts on wastewater treatment systems along the us coasts. Earth’s Future.

[46] Dehmamy N, Milanlouei S, Barabási AL (2018). A structural transition in physical networks. Nature.

[47] Bianconi G, Dorogovtsev SN (2014). Multiple percolation transitions in a configuration model of a network of networks. Phys. Rev. E.

[48] Karrer B, Newman ME, Zdeborová L (2014). Percolation on sparse networks. Phys. Rev. Lett..

[49] Mezard, M. & Montanari, A. *Information, Physics, and Computation* (Oxford University Press, 2009).

[50] Pei S, Teng X, Shaman J, Morone F, Makse HA (2017). Efficient collective influence maximization in cascading processes with first-order transitions. Sci. Repo..

[51] Davies, R. Catastrophic flooding in houston and south east texas. http://floodlist.com/america/usa/flooding-houston-south-east-texas-august-2017 (2017).

[52] Brakenridge, G. & Kettner, A. J. DFO flood event 4510. http://floodobservatory.colorado.edu/Events/2017USA4510/2017USA4510.html (2017).

[53] Barron, D. & Hill, G. A. These are the roads that are closed in Houston due to Hurricane Harvey. https://www.chron.com/news/houston-weather/hurricaneharvey/article/These-are-the-roads-that-are-closed-in-Houston-12003482.php (2017).

[54] National Oceanic and Atmospheric Administration. Post tropical cyclone harvey report. https://nwschat.weather.gov/p.php?pid=201803221510-KHGX-ACUS74-PSHHGX (2017).

[55] Li D (2015). Percolation transition in dynamical traffic network with evolving critical bottlenecks. Proc. Natl Acad, Sci. USA.

[56] Kahle, D. & Wickham, H. ggmap: A package for spatial visualization with google maps and openstreetmap. r package version 2.3. *R Foundation for Statistical Computing, Vienna* (2013).

[57] Yamazaki D, Kanae S, Kim H, Oki T (2011). A physically based description of floodplain inundation dynamics in a global river routing model. Water Resources Res..

[58] Achlioptas D, D’souza RM, Spencer J (2009). Explosive percolation in random networks. Science.

[59] Braunstein A, DallAsta L, Semerjian G, Zdeborová L (2016). Network dismantling. Proc. Natl Acad. Sci. USA.

[60] Warren CP, Sander LM, Sokolov IM (2002). Geography in a scale-free network model. Phys. Rev. E.

[61] Morone F, Makse HA (2015). Influence maximization in complex networks through optimal percolation. Nature.

[62] Lü L, Zhou T, Zhang QM, Stanley HE (2016). The h-index of a network node and its relation to degree and coreness. Nat. Commun..

[63] Cavallaro M, Asprone D, Latora V, Manfredi G, Nicosia V (2014). Assessment of urban ecosystem resilience through hybrid social–physical complex networks. Comput.-Aided Civil Infrastruct. Eng..

[64] Bianconi G (2014). Multilayer networks: dangerous liaisons?. Nat. Phys..

[65] Radicchi F (2015). Percolation in real interdependent networks. Nat. Phys..

[66] Albert R, Barabási AL (2002). Statistical mechanics of complex networks. Rev. Mod. Phys..

[67] Majdandzic A (2016). Multiple tipping points and optimal repairing in interacting networks. Nat. Commun..

[68] Barthélemy M (2011). Spatial networks. Phys. Rep..

[69] Cai W, Chen L, Ghanbarnejad F, Grassberger P (2015). Avalanche outbreaks emerging in cooperative contagions. Nat. Phys..

[70] Hopcroft JE, Tarjan RE (1973). Algorithm 447: efficient algorithms for graph manipulation. Commun. ACM.

