# Supplementary material for: Local floods induce large-scale abrupt failures of road networks
Source: Nat Commun. 2019 May 15;10:2114. doi: 10.1038/s41467-019-10063-w (PMC6520386; doi:10.1038/s41467-019-10063-w)
Supplement: Supplementary file 1 — Supplementary Information [file 41467_2019_10063_MOESM1_ESM.pdf]

Supplementary Information

Local floods induce large-scale abrupt failures of road networks

Wang *et al.*

Supplementary Figures

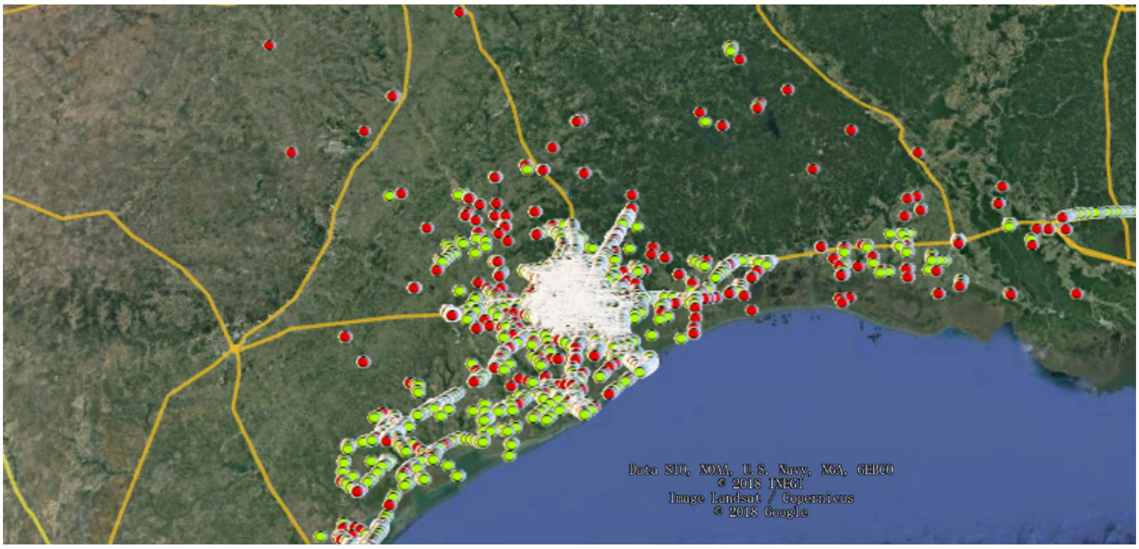

Supplementary Figure 1: **Mapping of failed road intersections resulted from Hurricane Harvey of 2017.** Red and green respectively indicate direct and indirect failures of road intersections.

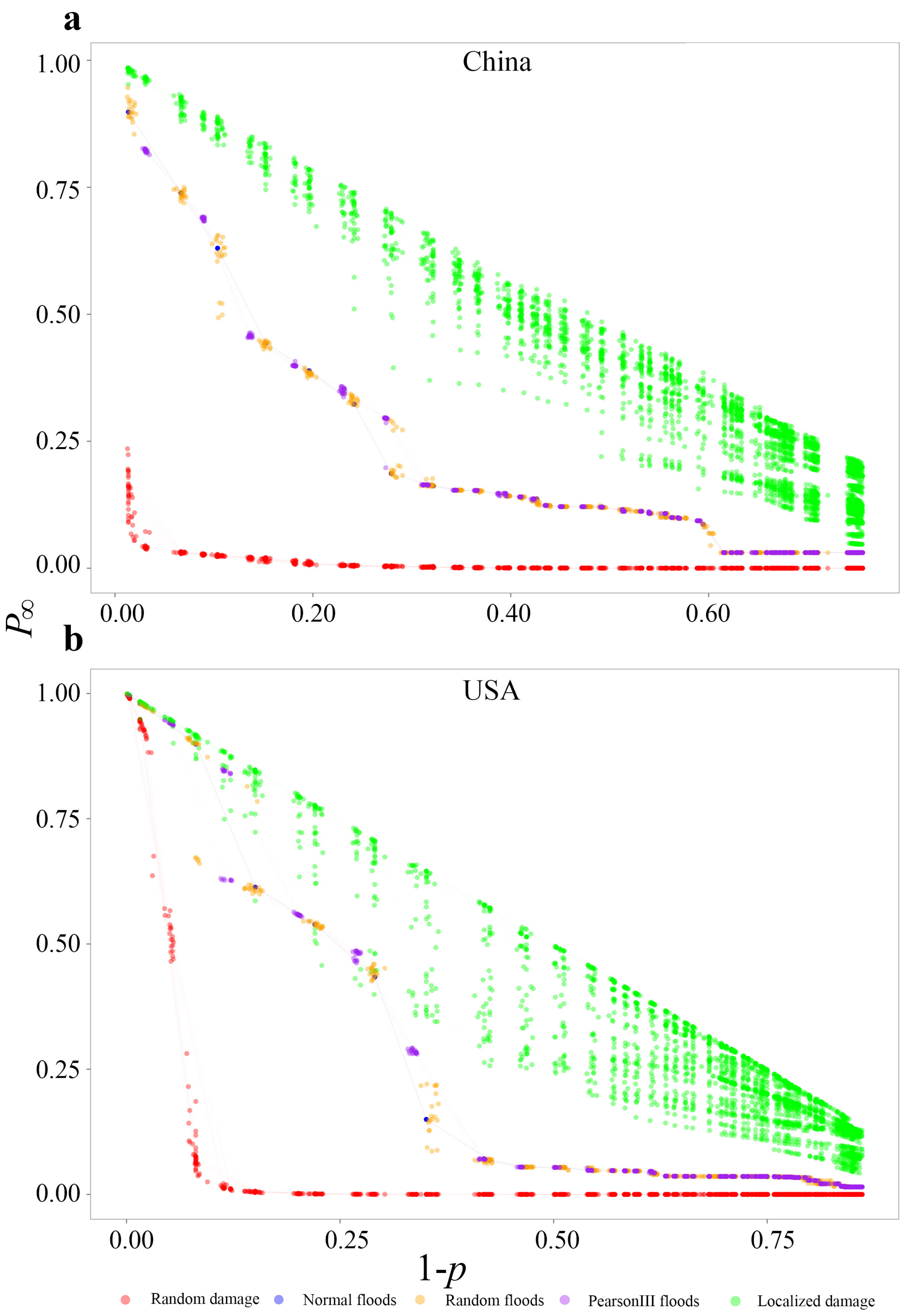

Supplementary Figure 2: **The fraction of nodes in the giant connected component of road network as a function of the fraction of direct failures.** The fraction of nodes in the giant connected component (P_∞) on the road network of China (a) and USA (b) as a function of direct failures ($1-p$) under floods, random damage and localized damage. Simulation results are the outcome of 20 independent runs.

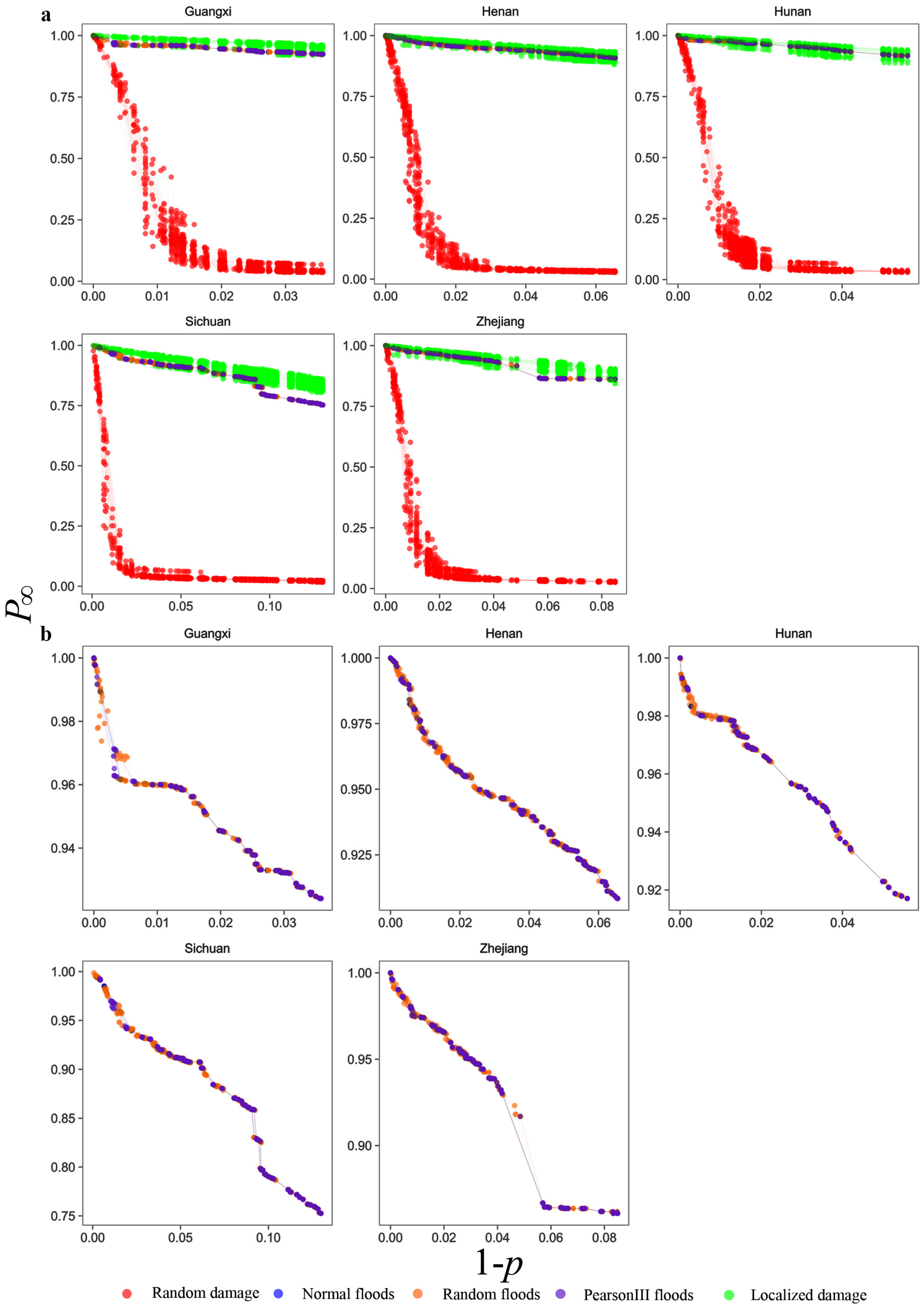

Supplementary Figure 3: **The fraction of nodes in the giant connected component on the road network of China as a function of the fraction direct failures.** (a) The fraction of nodes in the giant connected component (P_∞) on the road network of China as a function of the fraction of direct failures $(1-p)$ when each province is disturbed by floods, random damage and localized damage; (b)P_∞ on the road network of China as a function of $(1-p)$ when each state is disturbed by floods in different scenarios. Simulation results are the outcome of 20 independent runs.

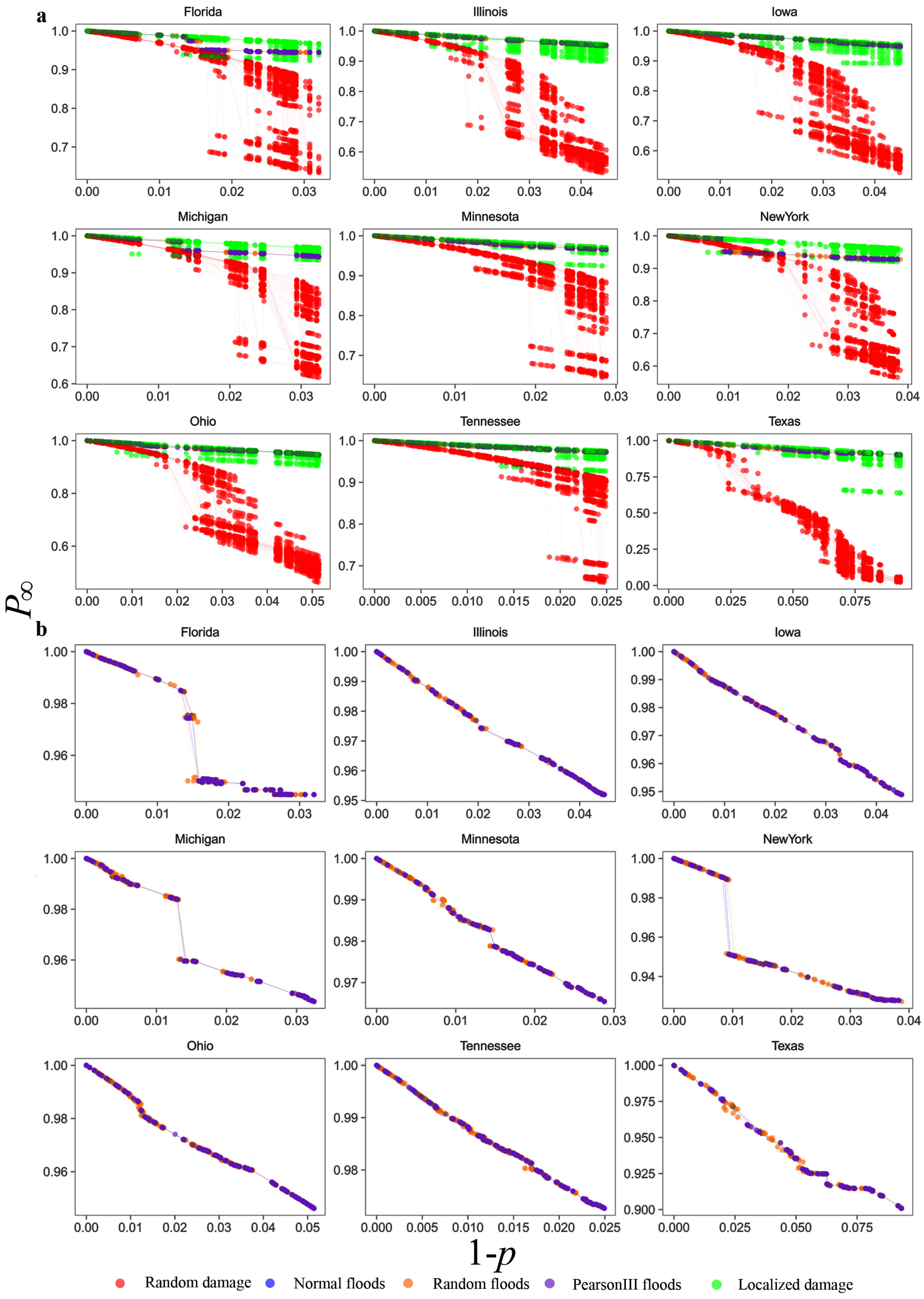

Supplementary Figure 4: **The fraction of nodes in the giant connected component (P_∞) on the road network of USA as a function of the fraction of direct failures ($1 - p$).** (a) The fraction of nodes in the giant connected component (P_∞) on the road network of USA as a function of the fraction of direct failures ($1 - p$) when each state is disturbed by floods, random damage and localized damage; (b) P_∞ on the road network of USA as a function of ($1 - p$) when each state is disturbed by floods in different scenarios. Simulation results are the outcome of 20 independent runs.

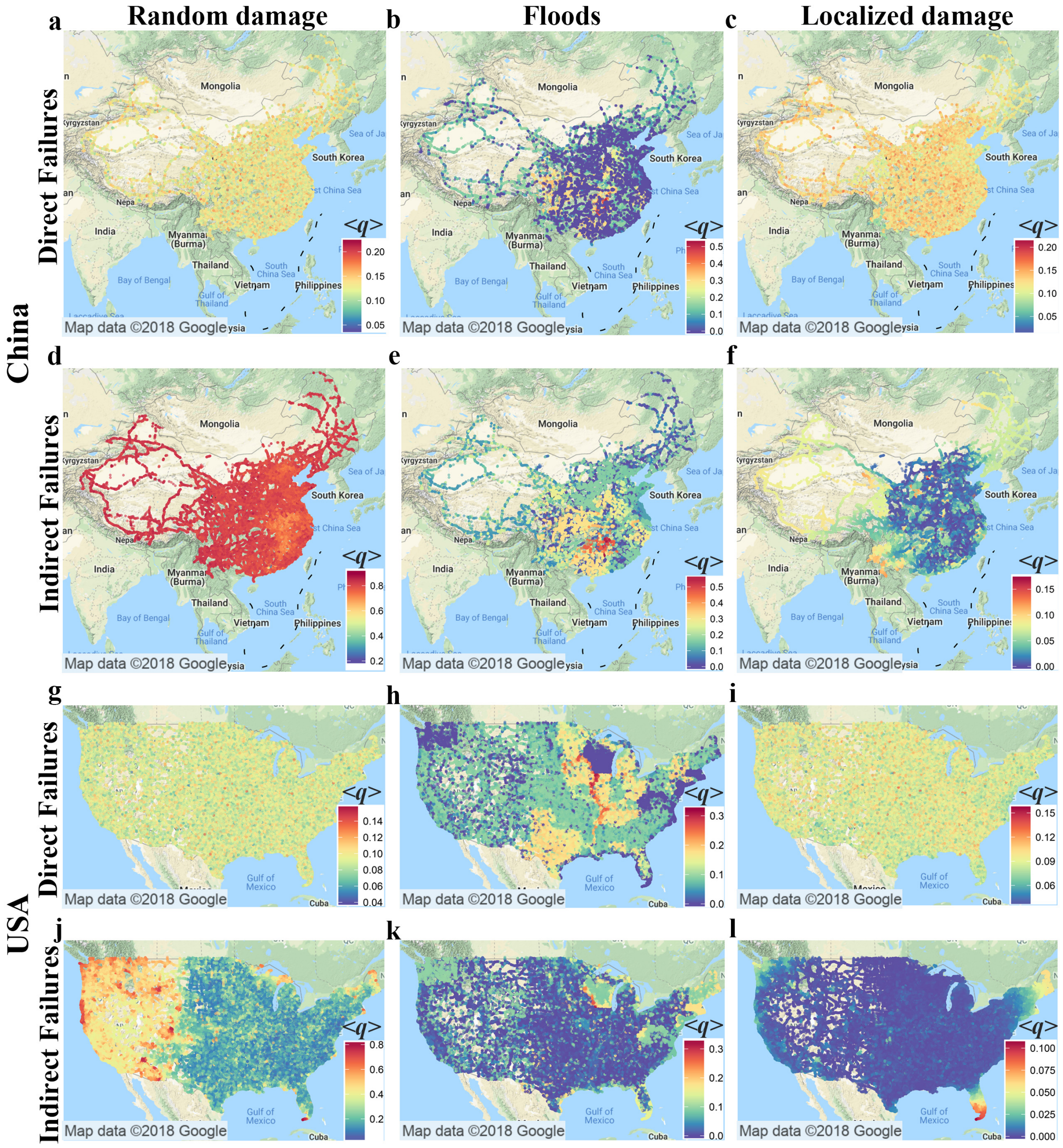

Supplementary Figure 5: **The aggregated vulnerability map of the road intersections in the USA.** Each road intersection is color-coded by the vulnerability to direct failures caused by random damage [a ($\langle q_i^{(d,r)} \rangle$)], floods [b ($\langle q_i^{(d,f)} \rangle$)] and localized damage [c ($\langle q_i^{(d,l)} \rangle$)]. Same as in (a, b and c) but color-coded separately for [d ($\langle q_i^{(i,r)} \rangle$), e ($\langle q_i^{(i,f)} \rangle$) and f ($\langle q_i^{(i,l)} \rangle$)] the vulnerability to indirect failures. The (g-l) are results in the US, respectively. The notations of aggregated vulnerability are shown in Table S1. We only consider road intersections in the giant connected component of the origin road network. Simulation results are the outcome of 20 independent global simulation runs and 20 regional simulation runs.

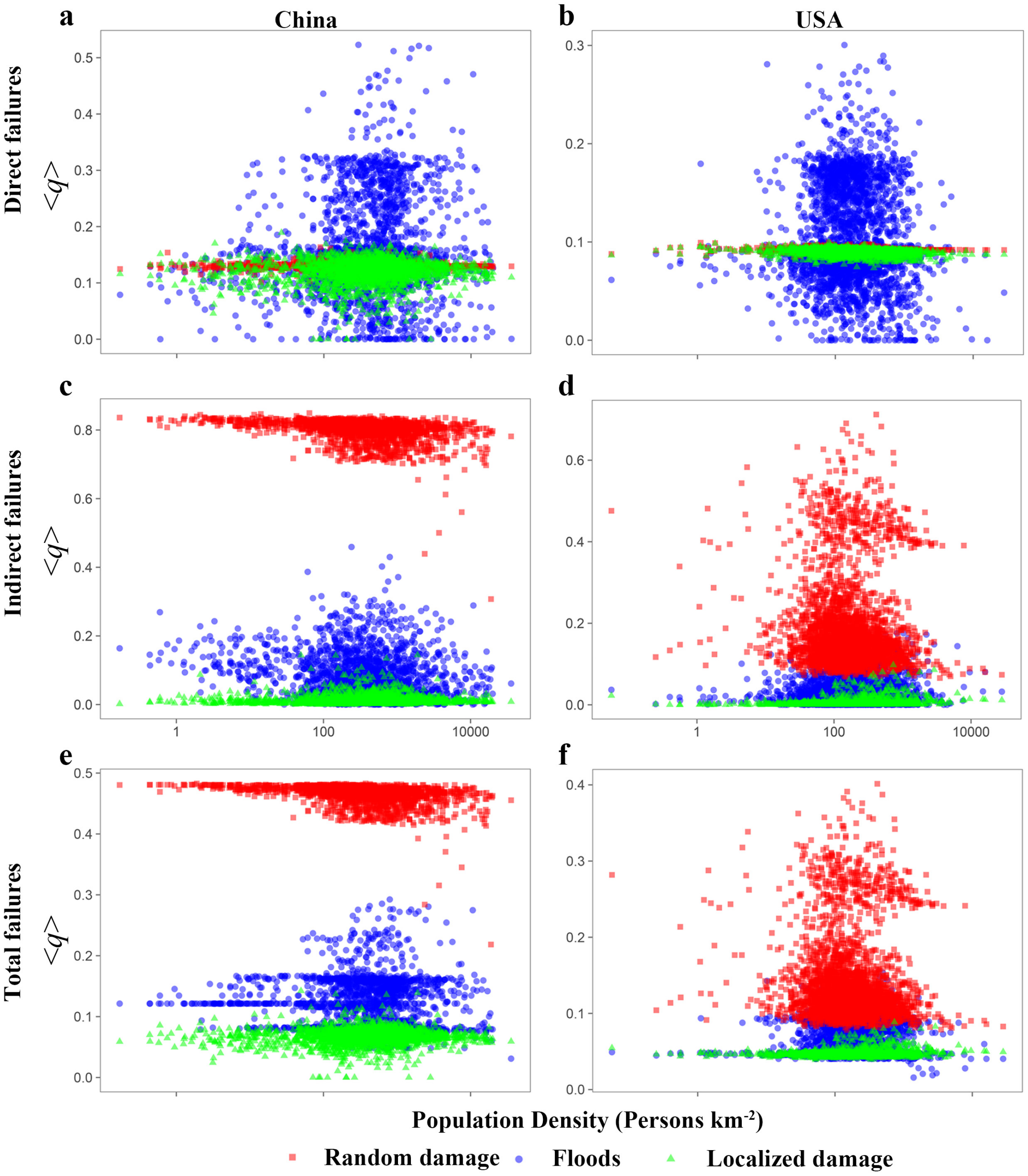

Supplementary Figure 6: **Vulnerability of counties with different population densities.** Population densities in China (a) and the USA (b) as a function of road intersections vulnerability to direct failures. The scatter points correspond to individual counties. For each county, the population density (in km^2) is calculated by the average population density of road intersections with $\langle q \rangle > 0$ within its boundary and the vulnerability is calculated by the average vulnerability of road intersections with $\langle q \rangle > 0$ within its boundaries. Population densities are from the Gridded Population of the World, Version 4 (GPWv4)[1]. The same as in (a) and (b) but shown separately for the vulnerability to indirect failures [China (c) and the USA (d)] and the vulnerability to total failures [China (e) and the USA (f)].

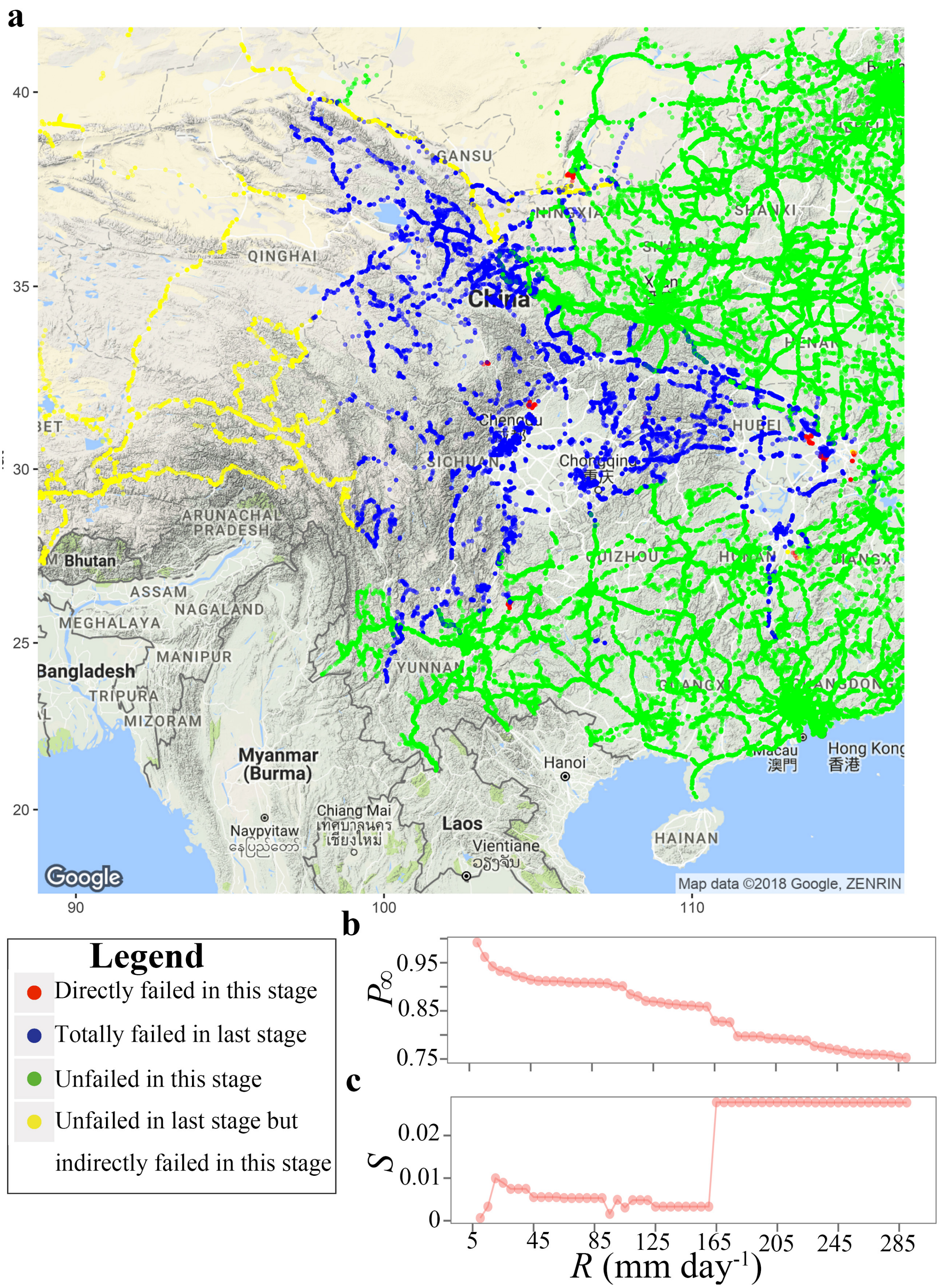

Supplementary Figure 7: **Percolation occurs in Sichuan.** (a) The geographical layout of road intersections disturbed by floods in Sichuan under the normal flood scenarios when input runoff (R) increases from 160mm (last stage) to 165mm (current stage) per day. (b) The fraction of nodes in the giant connected component (P_∞) of the road network of China as a function of runoff in Sichuan. (c) The fraction of nodes in the second largest connected component (S) of road network of China as a function of runoff in Sichuan.

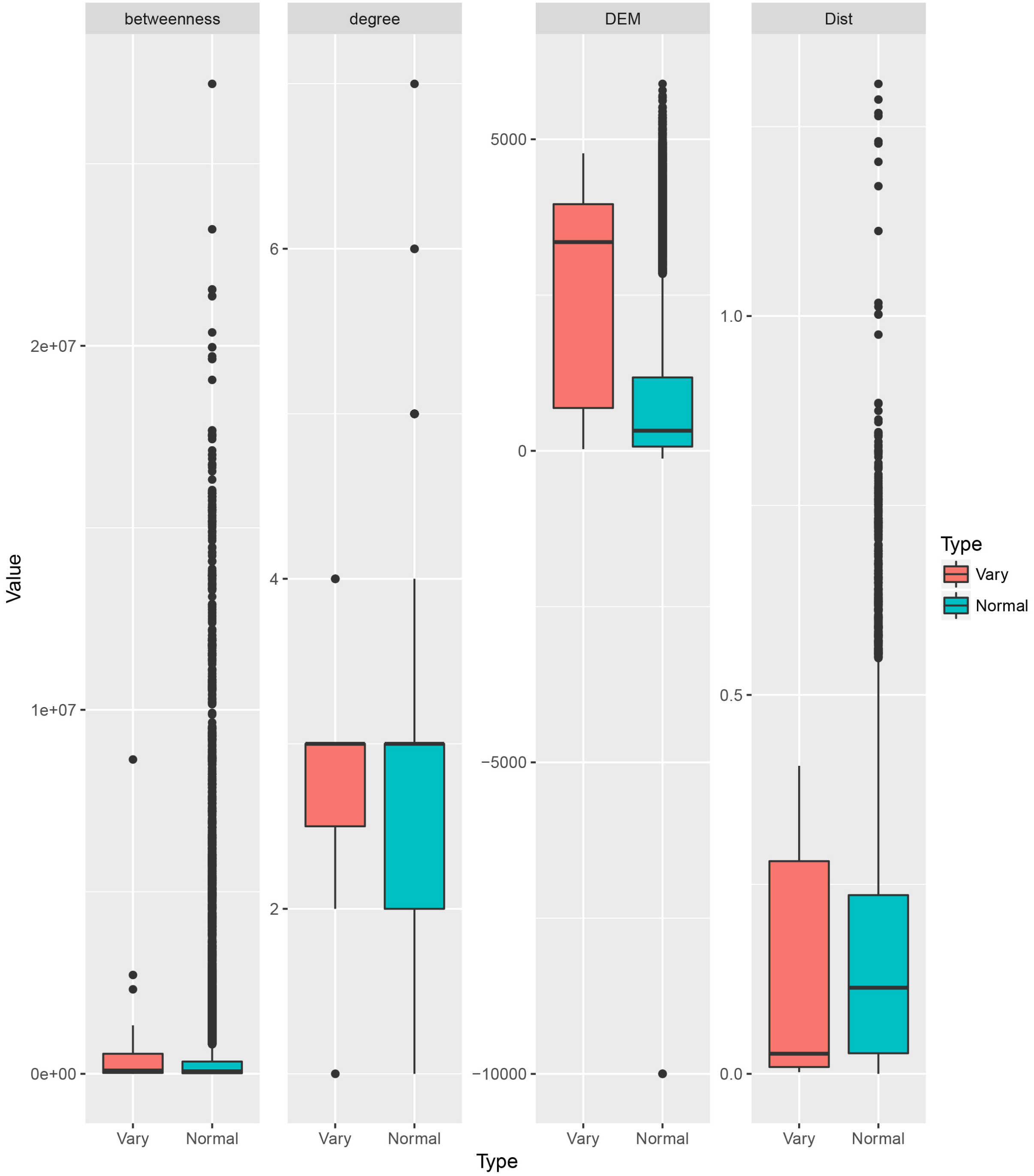

Supplementary Figure 8: **Boxplot of important measures: degree, altitude (DEM), betweenness and distance to river of the newly added inundated intersections (Vary) and all road intersections (Normal) in Sichuan.**

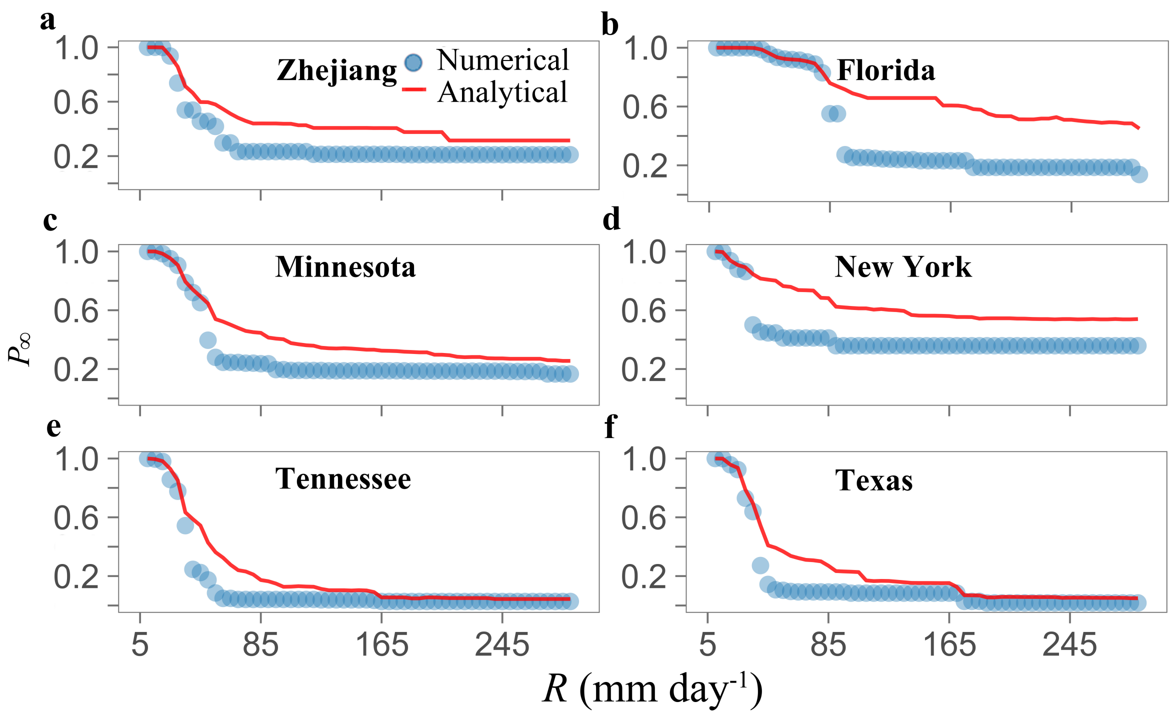

Supplementary Figure 9: **Comparison of numerical and analytical solutions.** (a) The fraction of nodes in the giant connected component (P_∞) of a road subnetwork in Zhejiang as a function of surface runoff (R). Blue points represent the numerical results while the red line represents the analytical results. (b–f) Giant connected component for road subnetworks as a function of surface runoff.

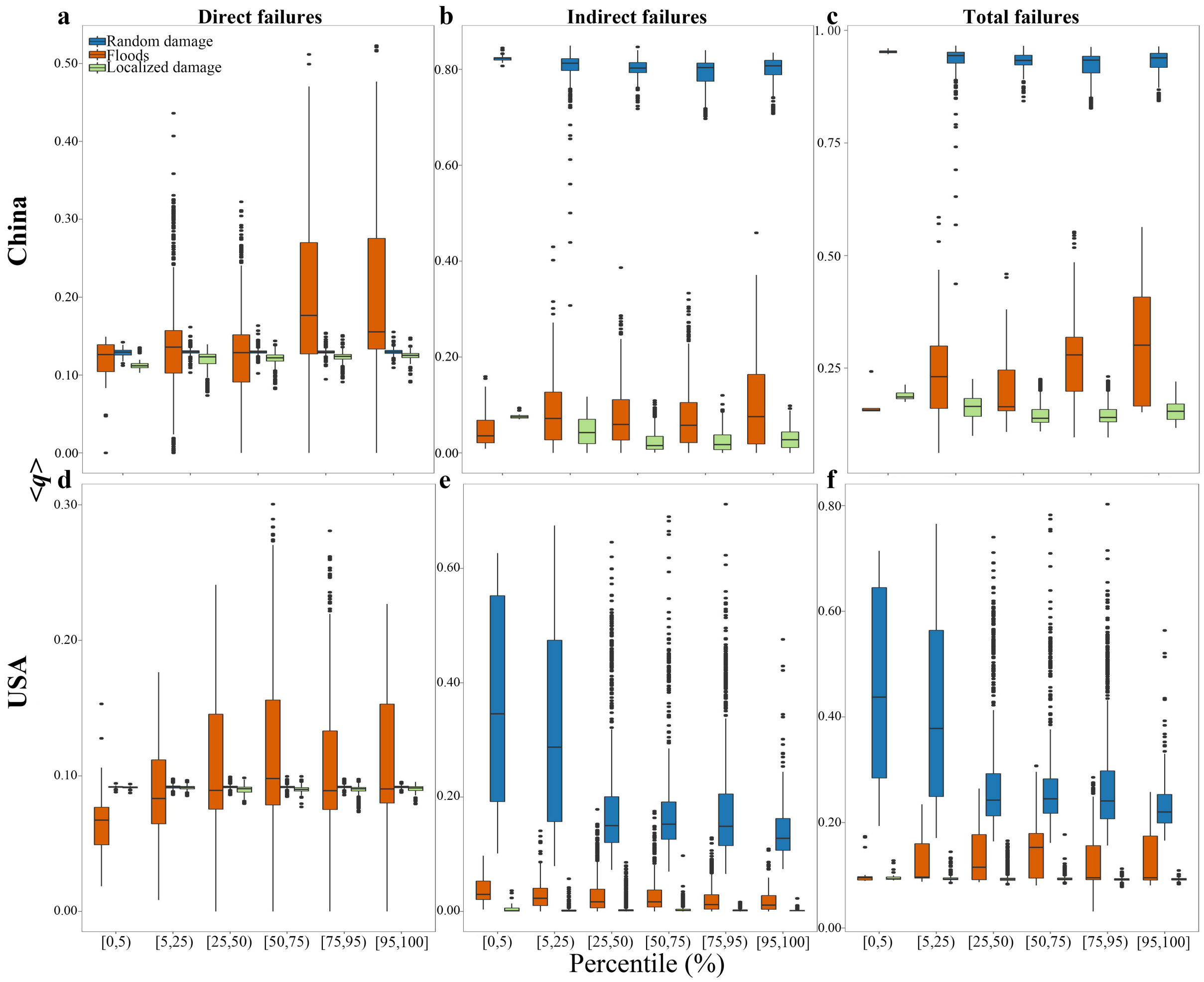

Supplementary Figure 10: **Vulnerability $\langle q \rangle$ of counties groups with different population densities.** Vulnerability to direct failures as a function of population density percentile for each county group in China (a) and the USA (d). The scatter points correspond to individual counties. For each county, the vulnerability and the population density percentile are computed by the average vulnerability and percentile of population density of road intersections with $\langle q \rangle > 0$ within its boundary, respectively. The vulnerability to indirect failures [China (b) and the USA (e)] and the vulnerability to total failures [China (c) and the USA (f)].

Supplementary Tables

Supplementary Table 1: The statistics of flooding damage description

Group	Description	$\lvert\mathcal{N}_i\rvert$	$F_i^{(t,f)}$	Counties
CN	catastrophic flooding occurred with numerous roads inundated	31,135	0.995810145	Galveston, Harris, Wharton, Montgomery
MF	major flooding occurred with few roads inaccessible	100	0.223713647	Madison, Matagorda, Walker
MM	major flooding occurred with many roads inundated	165	0.331991952	Polk, San Jacinto, Waller
MN	major/record flooding occurred with numerous roads inundated	5,481	0.944837097	Brazoria, Colorado, Fort Bend, Jackson, Lavaca, Liberty
MI	minimal impacts	31	0.058601134	Brazos, Trinity
MW	minor flooding occurred with few roads inaccessible	2	0.060606061	Lee
MO	moderate flooding occurred	7	0.104477612	Burleson
RR	moderate/record flooding occurred	175	0.77092511	Austin
NM	no impacts observed from the minor flooding	41	0.14137931	Grimes, Washington

Supplementary Table 2: Notation Summary.

Notation	Description
$\mathscr{B}$	Non-backtracking matrix
$\mathscr{C}$	The set of nodes in a connected component
$\mathbf{d} = (d_1, , d_{\lvert\mathscr{M}\rvert})$	The vector of ending node of edge in a network
$\mathscr{D}^{(f)}$	The set of removed nodes (direct failures) in a network resulted from floods
$\mathscr{D}^{(l)}$	The set of removed nodes (direct failures) in a network resulted from localized damage
$\mathscr{D}^{(r)}$	The set of removed nodes (direct failures) in a network resulted from random damage
$\mathbf{e} = (e_1, , e_{\lvert\mathscr{M}\rvert})$	The vector of edges in a network
f	The fraction of nonzero aggregated vulnerability
$F_i^{(t,f)}$	The ratio of total failures resulted from floods
$\mathscr{I}^{(f)}$	The set of indirect failures resulted from floods, the nodes (indirect failures) are disconnected from the giant connected component after removal of direct failures
$\mathscr{I}^{(l)}$	The set of indirect failures resulted from localized damage
$\mathscr{I}^{(r)}$	The set of indirect failures resulted from random damage
I	The fraction of nodes which are indirect failures in a network
k	The degree of a node in a network
$\mathscr{M}$	The set of edges in a network
$\mathbf{n} = (n_1, , n_{\lvert\mathscr{N}\rvert})$	The vector of binary failure status indicators for all nodes
$\mathscr{N}$	The set of nodes in a network
$\mathbf{o} = (o_1, , o_{\lvert\mathscr{M}\rvert})$	The vector of starting nodes of edges in a network
$1 - p$	The fraction of removed nodes (direct failures) in a network
$\mathscr{P}$	The set of nodes in the giant connected component
p_c	Critical (percolation) threshold
P_∞	The fraction of nodes in the giant connected component
$P(k)$	Degree distribution
$q_{i,j}$	the probability that a road intersection i fails during simulation j
$\langle q_i^{(d,f)} \rangle$	The aggregated vulnerability of the direct failures resulted from floods
$\langle q_i^{(d,r)} \rangle$	The aggregated vulnerability of the direct failures resulted from random damage
$\langle q_i^{(d,l)} \rangle$	The aggregated vulnerability of the direct failures resulted from localized damage
$\langle q_i^{(i,f)} \rangle$	The aggregated vulnerability of the indirect failures resulted from floods
$\langle q_i^{(i,r)} \rangle$	The aggregated vulnerability of the indirect failures resulted from random damage
$\langle q_i^{(i,l)} \rangle$	The aggregated vulnerability of the indirect failures resulted from localized damage
$\langle q_i^{(t,f)} \rangle$	The aggregated vulnerability of the total failures resulted from damage
$\langle q_i^{(t,r)} \rangle$	The aggregated vulnerability of the total failures resulted from random damage
$\langle q_i^{(t,l)} \rangle$	The aggregated vulnerability of the total failures resulted from localized damage
r	Correlation coefficient
R	Surface runoff
S	The fraction of nodes in the second largest connected component
$\mathscr{T}^{(c,f)}$	The set of reported failures which are covered by total failures
$\mathscr{T}^{(f)}$	The set of total failures resulted from floods
$\mathscr{T}^{(l)}$	The set of total failures resulted from localized damage
$\mathscr{T}^{(r)}$	The set of total failures resulted from random damage
$\mathscr{T}^{(r,f)}$	The set of road closures reported by TranStar and flooded streets (road segments) via public media (Barron and Hill, 2017) resulted from floods in Houston
v_i	The probability of node i belongs to the giant connected component

Supplementary Table 3: The size of region network

Region	Network Size	Region	Network Size	Region	Network Size
Guanxi	682	Florida	84,479	New York	59,887
Henan	3,211	Illinois	52,386	Ohio	48,148
Hunan	957	Iowa	19,063	Tennessee	20,707
Sichuan	2,293	Michigan	54,358	Texas	128,101
Zhejiang	11,464	Minnesota	31,889		

Supplementary Table 4: The different types of roads in our road data sets[2]

Code	Fclass	Description	OSM Tags
511x	Major roads		
5111	motorway	Motorway or freeway	highway=motorway
5112	trunk Important roads, typically divided	highway=trunk	
5113	primary Primary roads, typically national	highway=primary	
5114	secondary Secondary roads, typically regional	highway=secondary	
5115	tertiary Tertiary roads, typically local	highway=tertiary	
513x	Highway links (sliproads or ramps)		
5131	motorway_link	Roads that connect from one road to another of the same of lower category.	highway=motorway_link
5132	trunk_link		highway=trunk_link
5133	primary_link		highway=primary_link
5134	secondary_link		highway=secondary_link

Supplementary Notes

Supplementary Note 1: Three types of disturbance

The different behaviors of networks due to disturbances

The damage characteristics (behaviors) refer to giant component as a function of the fraction of removed nodes, also known as percolation theory [3].

When the fraction of removed nodes (direct failures) reaches a certain value $1 - p_c$, it leads to a percolation phase transition where the whole system will be completely fragmented and lose the function. This critical (percolation) threshold p_c indirectly reflects the robustness of a road network. The behaviors due to random damage are very different from that due to localized damage on a road network since road networks are spatially embedded 2-dimensional (or 3-dimensional) networks. Take the classic 2D lattice network for example, (1) $p_c = 0$ for localized damage, meaning that we have to remove all nodes from the lattice to destroy the network; (2)$p_c = 0.5927$ for random damage, meaning that we only need to remove 0.4073 fraction of all nodes randomly to destroy the network[4]. The behaviors of other non-spatially embedded networks vary a lot[5]: (1) Erdös-Rényi (ER) networks with Poisson degree distribution show the same behaviors due to both localized damage and random damage $p_c = \frac{1}{\langle k \rangle}$, where $\langle k \rangle$ is the average degree of a network; (2) Random regular (RR) networks with the same degree from each node are more robust to localized damage $p_c = (k_0 - 1)^{-\frac{k_0}{k_0-2}}$ than to random damage $p_c = \frac{1}{(k_0-1)}$, where k_0 denotes each node is randomly connected to k_0 other nodes; (3) Scale-free (SF) networks with power law degree distribution $P(k) \sim k^{-\lambda}$ are more robust to localized damage than to random damage when $\lambda > 3.825$ and the opposite is true when $\lambda < 3.825$.

Unique features of flood effect

Floods, as a new and realistic type of network disturbance introduced in this paper, are more locally destructive and has stronger effect on a neighborhood or community than random damage and is not as simple as localized damage since rivers may spread the damages from one location to other locations. Therefore, the destructive effect of floods is somewhere between random damage and localized damage. Three-dimension is a hallmark of some

types of network [6]. It is interesting that the 3-dimensional road network demonstrates major differences among flood disturbances and other damages (e.g. random damage, and localized damage). We take road altitudes into account to understand a road network's robustness to flood disturbances. In contrast, we only need to use a 2D road network to analyze its robustness to random damage and localized damage.

Supplementary Note 2: Affected population in China and the US

More highly populated counties will be affected by floods in China than that in the US.

To compare the population density of affected counties in China and the US, we use the percentile of gridded population density in the comparison to avoid the influence of huge population differences in these two countries (1.40 billions vs. 0.33 billions). Population density is taken from the Gridded Population of the World, Version 4 (GPWv4)[1]. The percentile of population density of each grid in each country is calculated respectively. We associate each road intersection to the nearest grid and the grid percentile is recorded as the intersection's percentile of affected population density. For each county, the vulnerability and percentile of population density are computed as the average vulnerability and population density percentile of all road intersections with $\langle q \rangle > 0$ within its boundary. We then categorize all counties into 5 groups based on population density percentile ([0, 5), [5, 25), [25, 50), [50, 75), [75 100]) in China and the US respectively. The vulnerability and population density percentile of five county groups in China and the US are shown in Supplementary Figure 10. The highly populated counties in China are more likely to be affected by floods than in the US, from the perspectives of direct failures, indirect failures and total failures.

Counties with extremely high population density ([95, 100] percentile) are more likely to be directly affected by floods in China.

As shown in Supplementary Figure 10(a), the vulnerability of county group with extremely high population density ([95, 100] percentile) to floods is significantly higher than that

to random damage and localized damage. This is indispensable when making investment in infrastructure systems. If we can effectively prevent direct physical damage due to floods, we can efficiently reduce the total population losses resulting from floods in China.

Flood mitigation will be more challenging in China than that in the US.

We usually adopt various measures to control inundation (direct failures) rather than indirect failures in order to mitigate flood risk. However, China will suffer more indirect failures and have more affected population in contrast to the US, as shown in Supplementary Figure 10(b, e).

Supplementary Note 3: Why CaMa-Flood model?

CaMa-Flood is a creditable global hydrodynamic model. It has been wildly used to simulate region and global river floods [7–11] and validated by various situ and satellite observations [12] and benchmark data sets [13] in major world river basins, such as Amazon Congo, Orinoco, Mississippi, and extreme events, including the 2007 Cyclone Sidr in Bangladesh [14]. This paper focuses on the failures of national scale road networks (of China and the US) due to floods with different intensities. With this purpose, the CaMa-Flood model is applied to produce different floods on large geological scales. More importantly, we attempt to introduce the flood model as the realistic perturbations in a network and develop the corresponding percolation theory, showing novel phase transition phenomena when compared with artificial perturbations. With different research objects, researchers can consider other hydrological models, such as LISFLOOD[15, 16] and PCR-GLOBWB[17] to produce different flooding scenarios.

Supplementary References

[1] Center for International Earth Science Information Network - CIESIN - Columbia University. Gridded Population of the World, Version 4 (GPWv4): Population Density (2016). URL https://doi.org/10.7927/H4NP22DQ.

[2] Ramm, F. *et al.* OpenStreetMap Data in Layered GIS Format. *Version 0.6* **7** (2011).

[3] Bunde, A. & Havlin, S. Fractals and disordered systems (1991).

[4] Berezin, Y., Bashan, A., Danziger, M. M., Li, D. & Havlin, S. Localized attacks on spatially embedded networks with dependencies. *Scientific reports* **5**, 8934 (2015).

[5] Shao, S., Huang, X., Stanley, H. E. & Havlin, S. Percolation of localized attack on complex networks. *New Journal of Physics* **17**, 023049 (2015).

[6] Dehmamy, N., Milanlouei, S. & Barabási, A.-L. A structural transition in physical networks. *Nature* **563**, 676–680 (2018).

[7] Hirabayashi, Y. *et al.* Global flood risk under climate change. *Nature Climate Change* **3**, 816–821 (2013).

[8] Yamazaki, D. *et al.* Analysis of the water level dynamics simulated by a global river model: A case study in the amazon river. *Water Resources Research* **48** (2012).

[9] Yoshikawa, S. *et al.* Illustrating a new global-scale approach to estimating potential reduction in fish species richness due to flow alteration. *Hydrology and Earth System Sciences* **18**, 621–630 (2014).

[10] Ward, P. J. *et al.* A global framework for future costs and benefits of river-flood protection in urban areas. *Nature climate change* **7**, 642 (2017).

[11] Emerton, R. *et al.* Complex picture for likelihood of enso-driven flood hazard. *Nature communications* **8**, 14796 (2017).

[12] Yamazaki, D., Kanae, S., Kim, H. & Oki, T. A physically based description of floodplain inundation dynamics in a global river routing model. *Water Resources Research* **47**, 1–21 (2011).

[13] Pappenberger, F., Dutra, E., Wetterhall, F. & Cloke, H. L. Deriving global flood hazard maps of fluvial floods through a physical model cascade. *Hydrology and Earth System Sciences* **16**, 4143–4156 (2012).

[14] Ikeuchi, H. *et al.* Compound simulation of fluvial floods and storm surges in a global coupled

river-coast flood model: Model development and its application to 2007 c yclone s idr in b angladesh. *Journal of Advances in Modeling Earth Systems* **9**, 1847–1862 (2017).

[15] Bates, P. D. & De Roo, A. A simple raster-based model for flood inundation simulation. *Journal of hydrology* **236**, 54–77 (2000).

[16] Van Der Knijff, J., Younis, J. & De Roo, A. Lisflood: a gis-based distributed model for river basin scale water balance and flood simulation. *International Journal of Geographical Information Science* **24**, 189–212 (2010).

[17] Winsemius, H. *et al.* A framework for global river flood risk assessments. *Hydrology and Earth System Sciences* **17**, 1871–1892 (2013).
